# A human self-stimulatory CD4^+^ T cell antigen-presenting cell subset presents and recognizes agonistic self-antigens to sustain exponential autologous clonal expansion

**DOI:** 10.1093/jimmun/vkag274

**Published:** 2026-10-21

**Authors:** Christian K Smallwood, Sunny L Sneed, Chandler L Williford, Tylar S Roof, Mark D Mannie

**Affiliations:** Department of Microbiology and Immunology, Brody School of Medicine, East Carolina University, Greenville, NC, United States; Department of Microbiology and Immunology, Brody School of Medicine, East Carolina University, Greenville, NC, United States; Department of Microbiology and Immunology, Brody School of Medicine, East Carolina University, Greenville, NC, United States; Department of Microbiology and Immunology, Brody School of Medicine, East Carolina University, Greenville, NC, United States; Department of Microbiology and Immunology, Brody School of Medicine, East Carolina University, Greenville, NC, United States

**Keywords:** antigen presentation/processing, human, MHC, T cell receptors, T cells

## Abstract

A new human xeno-free T cell expansion strategy enabled T cell receptor α/β (TCRα/β)–driven autologous reactivity and continuous long-term IL-2–dependent expansion of an unconventional professional T cell antigen-presenting cell (T-APC) subset. Autologous T-APC cultures were obtained from small peripheral blood mononuclear cell samples that yielded over 10^20^-fold expansions by an approach that did not require cell purification or genetic manipulation. Primary T-APCs constitutively expressed major histocompatibility complex class II glycoproteins, costimulatory B7 molecules, and FOXP3 in long-term culture. Autologous expansion yielded both CD4^+^ and CD8^+^ T-APC subsets that expressed signature activation markers and rapid cell division over 1 to 2 mo of culture in the absence of exogenous mitogenic or antigenic stimuli. Autologous IL-2–driven expansion of CD4^+^ T-APCs required TCR engagement because continuous T cell proliferation was blocked by neutralizing monoclonal antibodies against CD4, and constitutive expression of CD40L was inhibited by neutralizing major histocompatibility complex class II monoclonal antibodies. Long-term expansion required continuous TGF-β exposure, which increased HLA-DR and CD25 expression with consequent enhancement of antigen presentation, autologous TCR recognition, and IL-2 responsiveness. Low IL-2/TGF-β concentrations favored CD25^high^ CD69^low^ PD1^low^ T-APC subsets, whereas high IL-2/TGF-β concentrations favored CD25^low^ CD69^high^ PD1^high^ T-APC subsets. T-APC presentation of agonistic self-antigens induced extensive blastogenesis of polyclonal syngeneic responder T cells, some of which differentiated de novo into exponentially expanding T-APC lines. Overall, this study reveals professional T-APC subsets that exhibit exponential autologous expansion in vitro via autologous self-presentation and agonistic TCR self-recognition. These T-APCs may regulate homeostatic self-reactivity of the human TCRα/β repertoire and may mediate, in part, autologous mixed lymphocyte reactions.

## Introduction

Development and maintenance of the T cell receptor α/β (TCRα/β) T cell repertoire require TCR-mediated recognition of complexes comprising polymorphic major histocompatibility complex class I (MHCI) and major histocompatibility complex class II (MHCII) glycoproteins loaded with self-peptides (self-pMHCI and pMHCII ligands) that are presented by professional antigen-presenting cells (APCs).^1–10^ Via these self-recognition mechanisms, a clonotypically diverse T cell repertoire is selected by TCR recognition of diverse self-peptides of the immunological “self-peptide” peptidome.^11–13^ During positive thymic selection for example, approximately 1% to 5% of CD4^+^ CD8^+^ double-positive thymocytes specifically recognize self-pMHC ligands with intermediate efficacy, and these homeostatic TCR interactions elicit survival, maturation, and migration of T cells to extrathymic peripheral tissues. These positively selected self-reactive mature T cells constitute the conventional T cell (Tcon) repertoire that provides host defense against infectious pathogens. Other positively selected CD4^+^ CD8^+^ thymocytes recognize pMHC ligands with efficacies in a higher overlapping bandwidth, and these thymocytes differentiate into CD4^+^ FOXP3^+^ regulatory T cells (Tregs) to maintain peripheral tolerance by inhibiting pathogenic inflammation.^14–17^ Mature Tcons and Tregs perform immune surveillance as tissue-resident T cells or by cycling between nonlymphoid tissues and lymphoid tissues, in which T cells continuously scan peripheral APCs for cognate pMHC ligands.^18–24^ T cells selected by recognition of positively selecting pMHC ligands in the thymus continue to recognize identical or mimicry self-pMHC ligands in peripheral tissues, and these clonotypically specific TCR/self-pMHC interactions are required for T cell survival. By these mechanisms, diverse self-pMHCI and pMHCII complexes in peripheral tissues maintain clonotypically diverse CD8^+^ and CD4^+^ repertoires, respectively. Immunological self-recognition is also a feature of memory-like subsets that depend on continuous interactions with self-pMHC complexes.^7^^,^^25–27^

Continuous imprinting of self-reactivity in thymic and peripheral tissues is fundamental to T cell functionality. Positive selection of conventional clonotypes with intermediate levels of homeostatic self-reactivity for a given self-pMHC complex substantially increases the likelihood that the same mature T cell clone will efficaciously cross-react with a foreign peptide bound to the same MHC gene product.^17^^,^^28^^,^^29^ Thus, the inherent self-reactivity of the T cell repertoire optimizes use of self-MHC molecules as antigen-presenting vehicles for efficacious recognition of foreign antigen, which enables optimal host defense via the inherited set of self-MHC molecules. Positive selection of Treg clones bearing relatively higher levels of self-reactivity ensures that Tregs experience higher levels of TCR signaling than Tcons in environments lacking foreign antigen.^30^ This differential self-reactivity represents an important mechanistic foundation by which Tregs exert dominant self-tolerance to preempt autoimmunity and pathogenic inflammation.

The autologous mixed lymphocyte reaction (AMLR) is an in vitro representation of homeostatic human T cell self-reactivity. AMLRs are based on the in vitro observation that human T cells, when cultured with syngeneic macrophages, dendritic cells (DCs), or B cells, exhibit increased proliferation, upregulated cytokine secretion, and induction of activation and adhesion molecules.^31–40^ AMLRs are distinguished from xeno- or allo-MLRs in that AMLRs are entirely driven by intrinsic TCR reactivity to self-pMHC complexes on professional APCs. The stimulatory mechanism is mediated by MHCII-restricted recognition of self-pMHC complexes and CD86/CD80-mediated costimulation.^41^ AMLRs are representative of responses implicated in homeostatic T cell survival and maintenance of TCR sensitivity rather than pathological autoimmunity.^42^ AMLR intensities are proportional to the intensity of inflammatory environments and are directly correlated with professional APC activation. Overall, AMLRs are considered beneficial adaptive responses reflecting the native interconnectivity of the human TCRα/β repertoire.

Given the inherent self-reactivity of the human T cell repertoire, we explored whether homeostatic APC/T cell interactions could sustain long-term human T cell expansion in vitro. This study provides evidence that specialized CD4^+^ and CD8^+^ MHCII^+^ human T cell subsets robustly present agonistic self-pMHC ligands to themselves in an autologous self-stimulatory circuit. These human T cell antigen-presenting cell (T-APC) subsets appear to express both agonistic self-pMHC ligands and self-reactive TCRs to drive sustained self-stimulatory growth in xeno-free media with 10^20^-fold yields in the absence of exogenous antigens or mitogens. Self-reactive TCRs were implicated in the autologous growth of CD4^+^ T-APCs because IL-2–driven proliferation was blocked by neutralizing monoclonal antibodies (mAbs) against CD4, and constitutive expression of CD40L was inhibited by an anti-MHCII mAb. The concepts of “agonistic” versus “homeostatic” pMHC self-antigens were respectively defined by the capacity to elicit blastogenic T cell growth versus the capacity to impart homeostatic survival signals. This difference may reside in unique antigen presentation and TCR recognition attributes of T-APCs rather than intrinsic activities of particular self-antigens. That is, agonistic versus homeostatic self-pMHC recognition may reflect specialized microenvironments rather than qualitative distinctions of antigen structure. Currently, mechanisms underpinning “agonistic self-antigens” are not understood.

Human T-APC expansion cultures, representing both CD4^+^ and CD8^+^ T cell subsets, exhibited nearly 100% viability without evidence of exhaustion or death over 1 to 2 mo of continuous exponential growth. CD4^+^ T-APCs exhibited multiple functions, including self-stimulatory growth, activation of polyclonal memory T cells, and the initiation of new T-APC lines. T-APCs also comprised distinct subsets in that low IL-2/TGF-β concentrations favored CD25^high^ CD69^low^ PD1^low^ subsets and high IL-2/TGF-β concentrations favored CD25^low^ CD69^high^ PD1^high^ subsets. These findings represent an advance for the field by showing that autologous agonistic self-presentation and TCR recognition of self-pMHCII complexes on the same T cells are drivers of sustained exponential AMLR-like proliferation in long-term cultures in the absence of canonical APCs. Overall, specialized unconventional CD4^+^ and CD8^+^ T-APC subsets may represent pivotal mediators of homeostatic immune regulation.

## Methods

### Recombinant proteins and cell lines

Recombinant rat TGF-β1 (TGF-β) was expressed and purified from a transfected stable HEK293F line as described in previous studies.^43^ Purified TGF-β was activated by 10 min at 70 °C, and each TGF-β preparation was verified for bioactivity by inhibition of granulocyte-macrophage colony-stimulating factor (GM-CSF)–stimulated TF-1 erythroleukemia cell growth (ATCC; CRL-2003).^44^ Recombinant human IL-2 was purified from a transfected stable HEK293F cell line, and bioactivity was assessed by proliferation of the IL-2–dependent murine SJL-PLP.1 T cell line.^45^ Recombinant human IL-2 was also obtained from a National Institutes of Health bioresource program (Teceleukin, Hoffman LaRoche) and from Thermo Fisher Scientific (PeproTech #200-02). Recombinant human IL-4 and GM-CSF were obtained from commercial vendors (Peprotech #200-04 and BioLegend #572903, respectively). GM-CSF and IL-4 bioactivity were assessed by proliferation of TF-1 cells. The murine BW5147 TCRα/β^−^ thymoma variant (Cellosaurus RRID: CVCL RW62)^46^ and the human SeAx T cell line (Cellosaurus entry CVCL 5363)^47^^,^^48^ were used as controls in proliferative assays. Phytohemagglutinin lectin from *Phaseolus vulgaris* (PHA or PHA-L) and concanavalin-A (Con-A) were purchased from Sigma-Aldrich.

### Derivation of human T-APC lines

Peripheral blood mononuclear cells (PBMCs) were obtained by density gradient centrifugation from the blood of healthy human volunteers who provided informed consent as approved by the University and Medical Center Institutional Review Board of East Carolina University (23-000339). PBMCs were cultured as nonfractionated populations or as purified CD4^+^ and CD8^+^ T cell subsets magnetically sorted by REAlease CD4 or CD8 Microbead Kits (Miltenyi Biotec #130-117-037 or #130-117-036). PBMCs were cultured in LymphoOne T-Cell Expansion Xeno-Free Media (Takara Bio #WK552S). During long-term expansion, T-APCs were passaged every 3 to 7 d with recombinant human IL-2 in the presence or absence of TGF-β. Both IL-2 and TGF-β are prototypic cytokines that drive FOXP3^+^ Treg differentiation and expansion pathways.^49–51^ T cells were maintained in vented-cap tissue culture flasks in standard CO_2_ incubators. Three approaches were used to derive T-APC lines, distinguished by whether PBMC cultures were initiated with (i) mitogen, (ii) an AMLR, or (iii) established T-APCs. The commonality was that T-APCs were expanded during the exponential growth phase by culture in the presence of IL-2 with or without TGF-β. Standard concentrations for IL-2, IL-4, and GM-CSF were 1 nM, and standard concentrations for TGF-β were 1 to 10 nM as designated. A separate approach (d) was used to derive conventional T cells (i.e. Tcons/ConA blasts) which were used as responders in T-APC/Tcon cocultures (Table 1).

**Table 1:** Human T cell lines used in this study.

Category	Method	Media	Cytokines	Putative APCs for initiation	Theoretical considerations	Cell line designations
**Mitogen initiated T-APC lines**	Nonfractionated PBMCs or CD4- or CD8-purified PBMCs were stimulated with mitogen during the first culture and propagated thereafter without mitogen.	LymphoOne media (xeno-free media)	IL-4, GM-CSF, IL-2, TGF-β for initiation (∼2 wk); IL-2 and TGF-β for expansion (mo)	IL-4/GM-CSF–induced myeloid DCs	Mitogen may recruit nonphysiological clonotypes.	CL1, CL2, CL5, CL6, CL7, NL1, P1, P2, P3, and CL2.2G4
**Spontaneous T-APC lines generated by an AMLR**	Nonfractionated PBMCs or CD4- or CD8-purified PBMCs were cultured without mitogen or exogenous antigen.	LymphoOne media (xeno-free media)	IL-4, GM-CSF, IL-2, TGF-β for initiation (∼2 wk); IL-2 and TGF-β for expansion (mo)	IL-4/GM-CSF–induced myeloid DCs	Faithfully reflects AMLR responders	CL4, V1, V3, JL2.C5, JL2.C6, JW4
**T-APC conversion of Tcons into new T-APC lines**	Tcon and T-APC cocultures exhibited extensive cell death. After 2–3 wk, new T-APCs emerged and exhibited exponential growth.	LymphoOne media (xeno-free media)	IL-2 for initiation and expansion	Dying parental T-APCs	Faithfully reflects AMLR responders	JL3.B1, JL3.C2, JL3.T1
**Tcons**	PBMCs were stimulated during the initial culture with mitogen and IL-2 and thereafter propagated in the presence of IL-2 only.	10% FBS-supplemented cRPMI (xeno-replete media)	IL-2 for initiation and expansion	None	Memory-effector T cells	JL3 Tcons (JL3 Tcon1 vs Tcon2, etc. are equivalent JL3 sublines)

#### Mitogen-initiated lines

During the first passage, PBMCs were cultured with PHA (5 μg/mL) and IL-2 in the presence or absence of TGF-β. Mitogenic stimulation was not used after the first passage. During the initial ∼2 wk, GM-CSF and IL-4 were added to cultures based on the hypothesis that an expanded presence of myeloid DCs would facilitate self-antigen recognition which in turn would catalyze T-APC activation and differentiation. Previous studies have shown that the combination of GM-CSF and IL-4 are the key cytokines driving expansion and differentiation of DCs.^41^^,^^42^ Culture duration spanned several weeks to months. Distinct human FOXP3^+^ T-APC lines from multiple donors were derived via this approach, including the CL1, CL2, CL5, CL6, CL7, NL1, P1, P2, P3, and CL2.2G4 lines. The CL2.2G4 line was derived from the mitogen-initiated CL2 line during the exponential growth phase via single cell cloning at limiting dilution (0.5 cells/well) in the presence of 1 nM IL-2 and without exogenous antigens or mitogens.

#### Spontaneous T-APC lines initiated by an AMLR

This method was the same as mitogen-initiated lines with the exception that no mitogen was used. PBMCs were cultured with IL-4, GM-CSF, IL-2, and TGF-β for the initial 2 wk, and cultured thereafter with only IL-2 with or without TGF-β during the exponential expansion phase. Spontaneous, AMLR-generated T-APC lines included the CL4, V1, V3, JL2.C5, JL2.C6, and JW4 lines.

#### T-APC conversion of Tcons into new T-APC lines

Senescent nonproliferative JL2.C5 T-APCs were or were not added to 96-well plates at ½-log titrations ranging from 10^5^ to 6.25 × 10^3^ cells/well with or without rested quiescent JL3 Tcons (10^5^/well). On day 11, cultures were transferred to 24-well plates containing 1 mL fresh media and 1 nM IL-2. On day 18, cell samples were enumerated with 2.5 × 10^4^ CountBright Absolute Counting Beads (Invitrogen #36950) per sample. Cultures were maintained in 1 nM IL-2, and those eventually showing exponential blastogenic T-APC growth were passaged to culture flasks and maintained as exponentially growing T-APCs. These Tcon-originated T-APC lines include the JL3.B1, JL3.C2, and JL3.T1 lines.

#### Conventional T cells

Human PBMCs were activated with 2.5 μg/mL Con-A and 1 nM IL-2 for 3 to 5 d in xeno-replete complete RPMI media (cRPMI) (10% heat-inactivated fetal bovine serum, 2 mM glutamine, 100 μg/mL streptomycin, 100 U/mL penicillin, 50 μM 2-ME). Con-A blast cells were then propagated with 1 nM IL-2 at 1 to 5 × 10^5^ cells/mL with passages every 3 to 7 d until the cells exhibited a rested quiescent nonproliferative morphology.

### In vitro bioassays

To measure antagonistic activity of anti-CD4 mAbs, human T cells were cultured in LymphoOne media in 96-well plates with designated concentrations of IL-2 in the presence of anti-CD4 mAbs or controls (BioXCell) (10–20 μg/mL). These mAbs included the anti-CD4 non-neutralizing OKT4 (mouse IgG2b-κ, BE0003-2), the neutralizing RPA-T4 (mouse IgG1-kappa, BE0288), or the respective isotype controls (MPC-11 mouse IgG2b-κ, BE0086 or MOPC-21 mouse IgG1-κ, BE0083). Cultures were maintained for designated durations and were pulsed with 1 µCi [^3^H]thymidine (6.7 Ci/mmol; New England Nuclear, PerkinElmer) during the last 24 h of culture. Cultures were harvested onto filters by use of a Tomtec Mach III harvester.[^3^H]thymidine incorporation into DNA was measured by use of a PerkinElmer MicroBeta2 liquid scintillation counter.

To measure inhibition of CD40L expression, T cells were cultured with 1 nM IL-2 and/or TGF-β in 96-well plates at 5 to 10 × 10^4^ cells/well in the presence or absence of the anti-MHCII L243 mAb (BE0306), the anti-CD40L 5C8 mAb (BE0292), or the isotype control (C1.18.4 mouse IgG2a-κ, BE0085) (BioXCell) (10 μg/mL) for 1 to 2 d. During the last 2 h of culture, a 20 μL mastermix containing 0.5 to 1.0 μL of the PE-conjugated anti-CD40L 24-31 mAb (BioLegend) was added directly to the 96-well cultures, as described in Chattopadhyay et al.^52^^,^^53^ The cells were then processed for flow cytometric analysis.

To measure T-APC activity, JL3.C2 T-APCs were irradiated (5 Gy; MultiRad 350; Precision X-ray) and were cultured at designated densities with or without JL3 Tcons (5 × 10^4^/well) in the presence of 1 nM IL-2. Cultures were pulsed with 1 µCi [^3^H]thymidine during the last 48 h of 5-d culture. Alternatively, JL3.C2 T-APCs were or were not added (10^4^ cells/well) to cultures in the presence or absence of JL3 Tcons (10^5^ cells/well) with 1 nM IL-2. On day 5, cells were passaged to a 24-well plate with 1 nM IL-2. On day 9, cell samples were subjected to flow cytometric analysis, and the remaining cells were propagated with IL-2 in flasks until day 24.

### Flow cytometric analysis

Cultured T cells were washed twice in phosphate-buffered saline (PBS) and stained with Live/Dead Fixable Blue Dead Cell Stain Kit (Thermo Fisher Scientific #L23105) for 30 to 60 min at room temperature in the dark. Cells were extensively washed and then incubated in Cell Staining Buffer (PBS + 2% bovine serum albumin + 1% human serum) for 30 min to remove unincorporated dye. For surface staining, cells were incubated with Fc Receptor Binding Inhibitor (Invitrogen #14-9161-73; 20 μL/test) for 20 min to block Fc receptors and nonspecific binding sites. Without washing, cells were stained with mAb cocktails in the dark at 4 °C for 4 h with rocking (200 μL volume). Intracellular staining was performed with the FOXP3 Intracellular Staining Kit (Invitrogen #00-5523-00). Specifically, cells were washed in PBS and resuspended in fixation/permeabilization solution (3:1 parts dilutant to concentrate) and were incubated in the dark for 30 min at room temperature. Cells were washed twice in permeabilization buffer and were incubated with designated mAbs in permeabilization buffer in the dark at 4 °C overnight with rocking (200 μL/tube). Cells were washed 3 times in permeabilization buffer and with diluted flow staining buffer (PBS + 0.2% bovine serum albumin + 0.1% Human Serum). Cells were analyzed on a Cytek Aurora Spectral Flow Cytometer (5-Laser Configuration) with unmixing via unstained and single-stained control samples followed by analysis with De Novo Software FCS Express 7. The following mAbs were used for staining (Table 2).

**Table 2:** mAbs used for flow cytometric analyses.

Marker	Fluorochrome	Clone	Company	Catalog #
**BCL6**	AF647	K112-91	BD Biosciences	561525
**CD3ε**	NovaFluor Blue 610-70S	SK7	Thermo Fisher Scientific	H028T03B06
**CD3ε**	PerCP-Cy5.5	HIT3a	BioLegend	300328
**CD3ε**	BV785	SK7	BioLegend	344842
**CD3ε**	BV510	SK7	BioLegend	344828
**CD4**	BV421	OKT4	BioLegend	317434
**CD4**	BUV496	SK3	BD Biosciences	612936
**CD8**	AF700	SK1	BioLegend	344724
**CD8**	Spark Blue 574	SK1	BioLegend	344784
**CD11c**	BV785	Bu15	BioLegend	337251
**CD19**	NovaFluor Yellow 730	HIB19	Thermo Fisher Scientific	H004T02Y07
**CD25**	APC/Fire 750	BC96	BioLegend	302642
**CD25**	PE/Fire 700	M-A251	BioLegend	356146
**CD25**	FITC	M-A251	BioLegend	356106
**CD25**	PE	M-A251	BioLegend	356104
**CD28**	BV510	CD28.2	BioLegend	302935
**CD28**	BV605	CD28.2	BioLegend	302968
**CD69**	PE	FN50	BioLegend	310905
**CD69**	APC/Fire 750	FN50	BioLegend	310946
**CD74 (Invariant chain)**	PE	LN2	BioLegend	326807
**CD80 (B7.1)**	BV711	2D10	BioLegend	305235
**CD86 (B7.2)**	BV650	2331 (FUN-1)	BD Biosciences	563412
**CD86 (B7.2)**	BV650	IT2.2	BioLegend	305428
**CD95 (FAS)**	BUV737	DX2	BD Biosciences	612790
**CD95 (FAS)**	PerCP/Fire 806	DX2	BD Biosciences	612790
**CD123 (IL3Rα)**	PerCP/Fire 806	S18016C	BioLegend	396615
**CD127 (IL7Rα)**	PE-Cy5	A019D5	BioLegend	351323
**CD134 (OX40)**	PE-Cy5	ACT35	BioLegend	350041
**CD152 (CTLA4)**	AF647	BN13	Thermo Fisher Scientific	MA5-44148
**CD154 (CD40L)**	PE	24-31	BioLegend	310806
**CD178 (FASL)**	BUV615	NOK-1	BD Biosciences	752306
**CD185 (CXCR5)**	BV605	MU5UBEE	Thermo Fisher Scientific	406-9185-41
**CD223 (LAG3)**	BUV563	3DS223H	Thermo Fisher Scientific	365-2239-41
**CD273 (PDL2)**	PE-Cy7	MIH18	BioLegend	345511
**CD274 (PDL1)**	APC eFluor780	MIH1	Thermo Fisher Scientific	47-5983-42
**CD279 (PD1)**	PE-Cy5	EH12.2H7	BioLegend	329971
**CD357 (GITR)**	BV711	108-17	BioLegend	371211
**FOXP3**	AF488	PCH101	Thermo Fisher Scientific	53-4776-42
**FOXP3**	AF488	150D	BioLegend	320012
**GARP/LRRC32**	PE-Cy7	7B11	BioLegend	352507
**HLA-A2**	NovaFluor Yellow 730	BB7.2	Thermo Fisher Scientific	H070T03Y07
**HLA-A, B, C**	Spark NIR 685	W6/32	BioLegend	311454
**HLA-DR**	BUV395	G46-6	BD Biosciences	564040
**HLA-DR**	Spark PLUS UV395	L243	BioLegend	307664
**HLA-DR, DP, DQ**	Spark Blue 574	Tu39	BioLegend	361724
**TCRα/β**	PE/Cy7	IP26	BioLegend	306719

### Statistical analyses, data presentation, and experimental reproducibility

Comparisons among 3 groups or more were assessed via 1-way or 2-way analysis of variance with the Tukey’s or Sidak’s multiple comparisons test (or as designated). Comparisons between two groups were analyzed by Student’s *t* test. A *P* value* <*0.05 was considered significant. Significance was indicated as follows: *P < 0.05, **P < 0.01, ***P < 0.001, and ****P < 0.0001. Each data point represents the mean value, and error bars represent the standard deviation. Experiments shown in the figures are representative of 3 independent experiments.

## Results

### Continuous TGF-β exposure augmented CD25 expression and enabled superior IL-2–driven growth of long-term FOXP3^+^ T cell cultures

Continuous TGF-β exposure of purified human CD4^+^ T cells in 38-d IL-2 expansion cultures enhanced cell enlargement (forward scatter mean values, Fig. 1A, B) compared with control T cells that were cultured without TGF-β. TGF-β–cultured T cells also exhibited higher expression of HLA-DR and CD25, lower expression of CD3, and steady expansion over the 38-d culture as assessed by 2-tailed unpaired *t* tests. Overall, these data provided evidence that continuous TGF-β signaling maintained T-APCs in an activated dividing state. These T cell lines were IL-2–dependent and were nearly 100% viable during exponential expansion. Consistent with this finding, a continuous 20-d exposure to TGF-β (i.e. since the initial derivation of the line) enabled high levels of IL-2–driven growth in proliferative bioassays, whereas control T cells (derived in the absence of TGF-β) lacked IL-2 reactivity at these cell densities (Fig. 1C). Significant differences (*P* < 0.0001, 2-way analysis of variance, Tukey’s multiple comparison test) existed in all group comparisons between TGF-β T cells versus control T cells at 10 pM, 100 pM, and 1 nM IL-2 for both nonfractionated PBMCs and purified CD4^+^ T cells. The main conclusion was that the continuous exposure to TGF-β augmented subsequent IL-2 reactivity. In contrast, TGF-β added directly to proliferative assays of long-term TGF-β–cultured T cells had modest inhibitory activity at 10 pM IL-2 (Fig. 1C, both left and right panels, *P* < 0.0001). These data are consistent with the possibility that TGF-β caused an increase in T-APC activity (i.e. higher HLA-DR) which in turn enhanced agonistic TCRα/β responses (lower CD3, higher CD25) to sustain high levels of IL-2 reactivity. Similar results were obtained with both nonfractionated T cells and purified CD4^+^ T cells (Fig. 1C, left and right, respectively).

**Figure 1:**
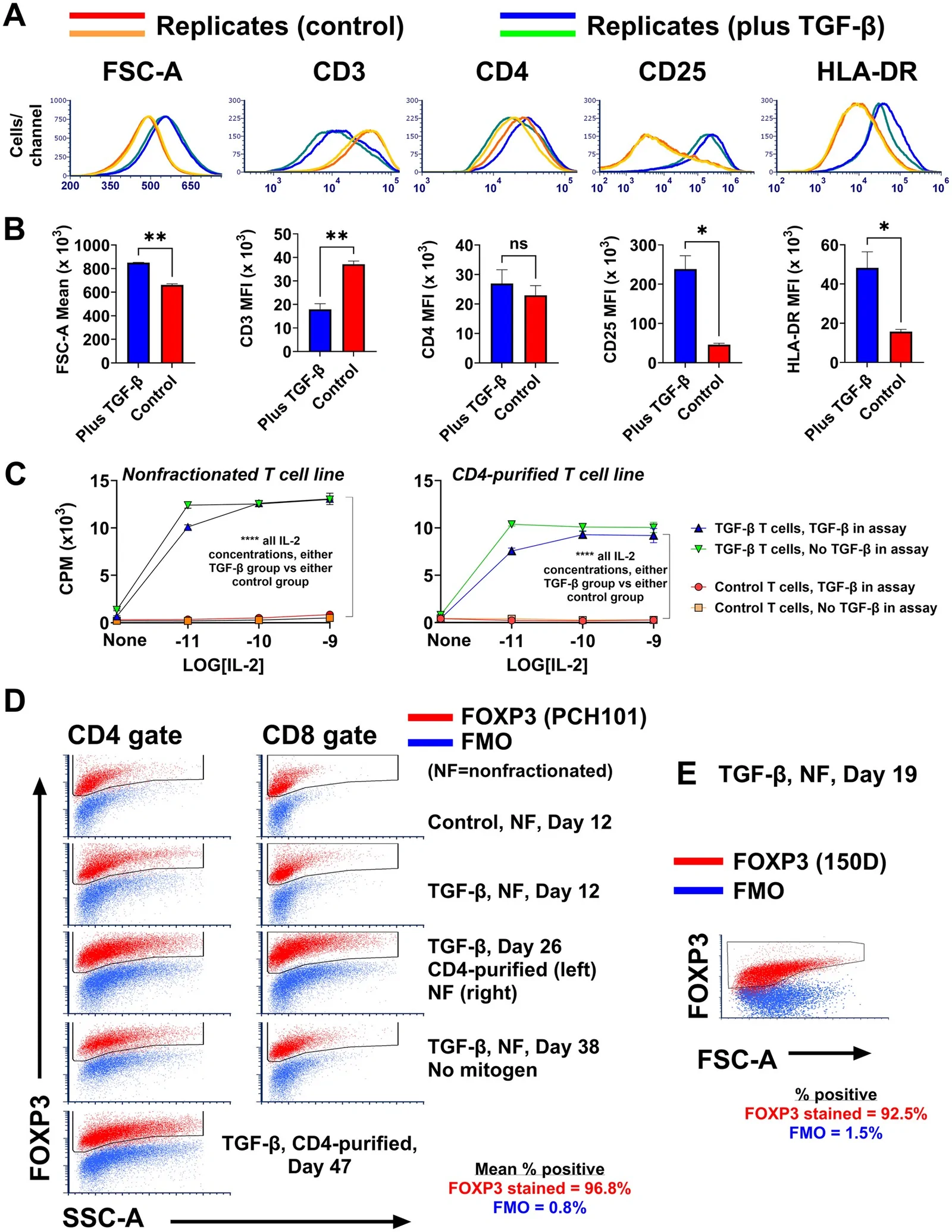
Continuous TGF-β exposure augmented CD25 expression and enabled superior IL-2–driven growth of long-term FOXP3^±^ T cell cultures. (A, B) CD4^+^ T cells were purified from PBMCs and were activated for 3 d with PHA and 1 nM IL-2 in the presence or absence of 10 nM TGF-β to initiate two long-term lines. TGF-β–cultured T cells and control T cells were then passaged every 3 to 7 d in 1 nM IL-2 with or without 10 nM TGF-β respectively for a total of 38 d. Cells were stained with anti-CD3 PerCP-Cy5.5 (HIT3a), anti-CD4 BV421 (OKT4), anti-CD8 AF700 (SK1), anti-CD25 PE (M-A251), and anti-HLA-DR BUV395 (G46-6). Histograms (A) and bar charts (B) show flow cytometric analyses of cell size (forward scatter) together with CD3, CD4, CD25, and HLA-DR expression (n = 2). (C) Nonfractionated PBMCs (left) and purified CD4^+^ PBMC T cells (right) were activated and maintained as in panel A for a total of 20 d. To assess IL-2 responsiveness in proliferative assays, TGF-β–cultured T cells and control T cells were incubated with 10 pM–1 nM IL-2 (x-axis) with or without TGF-β in flat-bottomed 96-well plates. Cells were pulsed with [^3^H]thymidine during the last 24 h of a 72-h culture (counts per minute [CPM]). (D) Independently derived T cell lines were initiated by a 3-d stimulation with PHA (rows 1, 2, 3, and 5) or without mitogen (autologous, row 4). T cells were cultured with 1 nM IL-2 in the presence or absence of 10 nM TGF-β (as designated) from day 0 until the day of analysis (day 12, 26, 38, or 47). Cells were stained with anti-FOXP3 clone PCH101 Alexa Fluor 488. (E) A nonfractionated T cell line was initiated as in panel A followed by continuous propagation in IL-2 and TGF-β for 19 d. Cells were gated on CD3^+^ and stained with the anti-FOXP3 clone 150D Alexa Fluor 488. Lines used in these experiments were CL2 (A, B), CL1 (C), NL1 (D, rows 1 and 2), CL1 (D, row 3), CL4 (D, row 4), CL2 (D, row 5), and P2 (E). *P < 0.05, **P < 0.01. FMO, fluorescence minus one; FSC-A, forward scatter area; ns, not significant.

Both CD4^+^ and CD8^+^ T-APC lines constitutively expressed FOXP3 (Fig. 1D). The CD4-gated/CD8-gated percentages were 42%/36% (top row), 52%/36% (second row), 94%/89% (third row), 47%/42% (fourth row), and 99%/<1% (fifth row), offset by small percentages of double-positive or double-negative (DN) subsets. Cells were stained with the anti-FOXP3 PCH101 clone that binds exon 1 and therefore can bind full-length FOXP3, the exon 2-spliced isoform (FOXP3Δ2), or the exon-2/exon-7 spliced isoform (FOXP3Δ2Δ7).^54^ Additional analyses with the anti-FOXP3 150D clone (Fig. 1E) also showed that a nonfractionated T-APC line (17% CD4^+^/62% CD8^+^) expressed FOXP3. This mAb binds exon 2 and thereby solely recognizes full-length FOXP3, which is known to be associated with optimal FOXP3 regulatory activity.^55–58^ Specificity of FOXP3 staining was compared with an FMO control (fluorescence-minus-one staining cocktails that lacked anti-FOXP3 mAb in the full panel). These data provide evidence that these long-term FOXP3^+^ T cell lines express the full-length form of FOXP3 but did not exclude coexpression of other spliced isoforms of FOXP3.

### Continual TGF-β exposure supported stable IL-2–dependent expansion of FOXP3^+^ T cells

To better understand the role of TGF-β in stable continuous T cell expansion, 3 PHA-stimulated PBMC cultures were propagated in 1 nM IL-2 in the presence or absence of 10 nM TGF-β for 80 d of continuous culture (Fig. 2A). T cells were passaged at approximately 10^5^/mL, and cell yields were tallied for each passage. Starting at day 0 with a theoretical value of a single T cell, over trillion-fold expansions were noted for TGF-β cultures over 80 d of culture, whereas T cell lines without TGF-β lost IL-2 responsiveness and viability after approximately 30 to 50 d of continuous culture. Early T-APC lines exhibited high levels of FOXP3 expression in both the presence and absence of TGF-β (Fig. 2B). Thus, T cell exposure to TGF-β was not required for FOXP3 expression, although by day 18, all TGF-β supplemented cultures had modestly higher levels of FOXP3 compared with control cultures without TGF-β (*P* = 0.0190, unpaired parametric 2-tailed *t* test of median values). On day 65, TGF-β expansion cultures comprised primarily CD8^+^ FOXP3^+^ T cells that expressed MHCII (HLA-DR), CD80, CD86, CD69, CD25, PD-L2 (CD273), PD1 (CD279), and CTLA4 (CD152) (Fig. 2C). These data indicate that TGF-β facilitates the long-term exponential growth of human T cells, with over a trillion to quadrillion-fold expansion potential. The day 80 time point was a chosen end of the experiment rather than a limitation of expansion potential. We conclude that this simple technology has the potential to provide large doses of human FOXP3^+^ T cells from small blood donations (20–40 mL) without cell purification.

**Figure 2:**
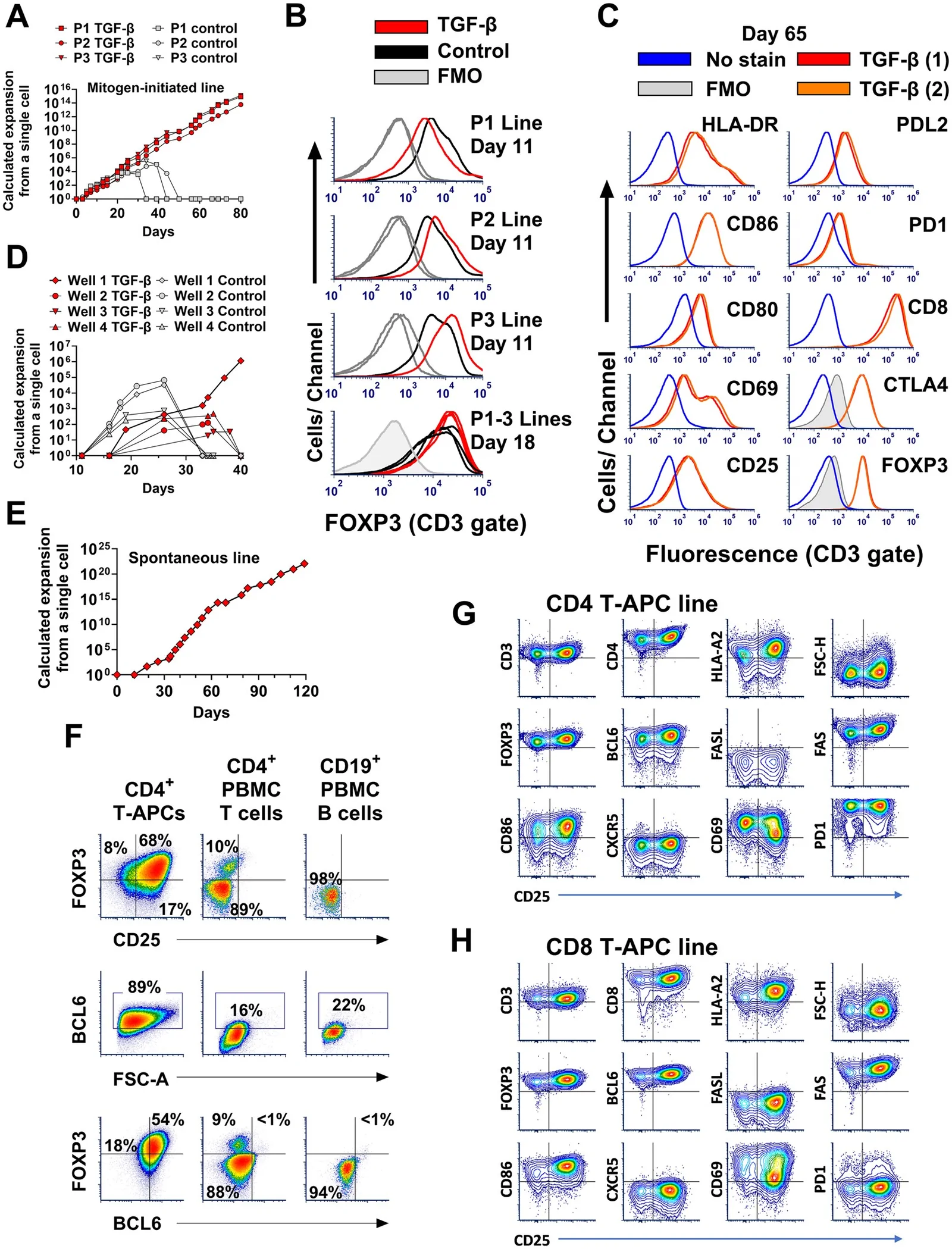
Continual TGF-β exposure supported stable IL-2–dependent expansion of FOXP3^±^ T cells. (A–C) PBMCs from 3 donors (P1, P2, and P3) were activated for 3 d with PHA and IL-2 in the presence or absence of 10 nM TGF-β. The respective cell lines were then propagated in IL-2 plus TGF-β versus IL-2 alone per the original culture conditions. Mitogens were not included after the first culture. Cells were passaged at equivalent densities of approximately 100,000 cells/mL. Day 0 represents purification and initiation of PBMC cultures, and each data point represents a cell count and a passage. Cell counts were recorded at each passage until the end of the experiment (day 80) or until cultures were lost due to cell death (A). The calculated expansion from a theoretical single cell (y-axis) is displayed against the number of days in culture (x-axis). The fold expansion for each passage was multiplied across all passages to calculate the total cell expansion from a theoretical single cell. Histograms show flow cytometric analysis on days 11 or 18 (B) and day 65 (C) after staining cells with CD3 NovaFluor Blue 610-70S (SK7), CD4 BV421 (OKT4), CD8 AF700 (SK1), CD25 APC-Fire750 (BC96), CD69 PE (FN50), CD80 BV711 (2D10), CD86 BV650 (FUN-1), CTLA4 AF647 (BN13), PDL2 PE-Cy7 (MIH18), PD1 PE-Cy5 (EH12.2H7), HLA-DR BUV395 (G46-6), and FOXP3 AF488 (PCH101). (D) Nonfractionated PBMCs from a healthy volunteer were seeded at 50,000 cells/well in LymphoOne medium in a flat-bottomed 96-well plate (n = 4) and cultured with 10 nM IL-2, 1 nM IL-4, and 1 nM GM-CSF with or without 10 nM TGF-β. Cells were passaged with new media and cytokines after approximately 7 d. Cell counts were initiated once growth was visually evident, and lines were passaged into T12.5 or T25 flasks for the remainder of experiment with 1 nM IL-2 with or without 10 nM TGF-β. IL-4 and GM-CSF were discontinued after 2 wk of culture. T cell expansion calculations were performed as in panel A. (E) The surviving T-APC line at day 40 in panel D is the same line portrayed in panel E (red diamonds). This culture exhibited sustained exponential growth through day 40 and was subsequently propagated through day 120 for a total 10^22^-fold expansion. (F) CD3^+^ CD4^+^ JL3.C2 T-APCs and human PBMCs (frozen stocks from a healthy human volunteer) were stained with CD3 BV510 (SK7), CD4 BV421 (OKT4), CD8 SB574 (SK1), CD19 StarBright UV795 (HIB19), CD25 APC-Fire 750 (BC96), FOXP3 Alexa Fluor 488 (150D), and BCL6 Alexa Fluor 647 (IG191E/A8). T-APCs were compared with CD3^+^ CD4^+^ and CD3^−^ CD19^+^ gated T and B cell PBMC subsets, respectively. (G, H) The phenotypes of a control CD4-purified T-APC line (day 33) and the CD8^+^ T-APC line from panel E (day 83) were assessed with CD3 NovaFluor Blue 610-70S (SK7), CD4 BV421 (OKT4), CD8 AF700 (SK1), CD25 APC-Fire750 (BC96), HLA-A2 NovaFluor Yellow 730 (BB7.2), FOXP3 AF488 (150D), BCL6 AF647 (K112-91), FASL BUV615 (NOK-1), FAS BUV737 (DX2), CD86 BV650 (FUN-1), CXCR5 BV605 (MU5UBEE), CD69 PE (FN50), and PD1 PE-Cy5 (EH12.2H7). Lines used in these experiments were P1, P2, and P3 (A–C), JL3.C2 (F), V1 (E, H), and V3 (G). FMO, fluorescence minus one; FSC-A, forward scatter area.

The expansion potential of autologous T cell cultures was evident from limited density cultures in which 50,000 nonfractionated PBMCs were seeded in the presence of IL-2 with or without TGF-β in the absence of mitogen (n = 4) (Fig. 2D). These data showed that exogenous TGF-β was not needed for autologous reactivity per se. Rather, initiation of autologous responses by control PBMC cultures preceded the onset of autologous growth of TGF-β–supplemented cultures. Thus, TGF-β delayed the onset of the autologous reaction. The benefit of TGF-β appeared to be an enablement of long-term, stable expansion during the IL-2–dependent exponential growth phase. The cell density of 50,000 cells/200 µL/well appeared to be a limiting dilution for T-APCs capable of long-term stable expansion (Fig. 2E). Whereas all cultures exhibited limited expansion during weeks 2 to 4, only 1 culture exhibited long-term exponential growth. This TGF-β–supplemented culture exhibited an approximate 10^22^-fold expansion during 3 mo of culture. These experiments provided evidence that most conventional T cells lacked the specialized attributes needed to differentiate into stable exponentially expanding T-APCs. Rather, these findings are most consistent with the view that only specialized precursors have the capacity to develop into long-term stable T-APC lines via a TGF-β–supported mechanism. This conclusion is consistent with the visual observations that after ∼10 d of culture, T-APC blasts appeared and outgrew the vast majority of small nonproliferative T cells.

To validate FOXP3 expression, a CD4^+^ T-APC line (JL3.C2) was compared with CD3^+^ CD4^+^ T cells and CD3^−^CD19^+^ B cells from healthy human volunteer PBMC samples (no culture, day 0) (Fig. 2F). CD4^+^ T-APCs were dominated by a single homogeneous FOXP3^+^ T cell population that also expressed high levels of CD25 (Fig. 2F, top left panel). T-APC FOXP3 levels were commensurate with levels expressed by peripheral blood CD4^+^ Tregs and qualitatively higher than baseline FOXP3 levels noted in peripheral Tcons or B cells. FOXP3^+^ T-APCs also expressed levels of the BCL6 transcription factor at levels that were qualitatively higher than baseline levels noted in PBMC T cells and B cells (Fig. 2F, 3 panels of 2nd row). These findings provide evidence that T-APCs are distinguished by homogenous expression of both FOXP3 and BCL6 (Fig. 2F, 3 panels of bottom row).

Because the P1, P2, and P3 nonfractionated lines spontaneously exhibited dominant outgrowth of CD8^+^ T cells, we used a purified control CD4^+^ T-APC line as a comparison for the CD8^+^ T-APC line (Fig. 2G). The CD4^+^ line expressed bimodal CD25^low^ and CD25^high^ populations along with high levels of CD3, CD4, MHCI (HLA-A2), FOXP3, BCL6, CD86, CD69, and PD1. Although this CD4^+^ line had high levels of PD1, other CD4^+^ and CD8^+^ T cell lines exhibited low PD1 expression. For example, the CD8^+^ T-APCs expressed high levels of CD3, CD8, MHCI, FOXP3, BCL6, CD86, and CD69 but lacked PD1 expression. The CD8^+^ line also expressed a dominant CD25^high^ subset along with minor CD25^low^ subset (Fig. 2H). Both CD4^+^ and CD8^+^ T-APC lines had high levels of CD95 (FAS) coupled with low levels of CD178 (FASL). These data showed that autologous expansion was a feasible approach to generate large FOXP3^+^ T cell populations from single 96-well PBMC cultures and that T-APC lines included a bimodal distribution of CD25^high^ and CD25^low^ subsets. These data also suggest that both CD4^+^ and CD8^+^ T-APCs may be subject to negative regulation by the FAS/FASL system.

### A neutralizing anti-CD4 mAb inhibited autologous MHCII-restricted autoantigen recognition and IL-2–dependent growth of CD4^+^ T-APCs

Given that CD4 is required for full TCR response potency but lacks known direct effects on the IL-2 receptor complex, we tested whether CD4 activity was needed for the IL-2–dependent blastogenic growth of CD4^+^ T-APCs. We focused on the CD4^+^ subset due to the commercial availability of defined neutralizing anti-human CD4 mAb reagents. Notably, the neutralizing anti-human CD4 mAb RPA-T4 but not the non-neutralizing OKT4 anti-CD4 mAb inhibited IL-2–stimulated growth of CD4^+^ FOXP3^+^ T cell lines (Fig. 3A). This inhibitory activity was specific for CD4^+^ T cells, because neither RPA-T4 nor OKT4 mAbs affected IL-2–dependent growth of CD8^+^ T cells (Fig. 3B). The RPA-T4 and OKT4 isotype controls lacked modulatory activity for both CD4^+^ and CD8^+^ T cells (Fig. 3A, B). None of these mAbs affected the IL-2–dependent growth of the mouse SJL-PLP.1 T cell line (Fig. 3C). The RPA-T4 and OKT4 mAbs bind distinct epitopes in the D1 and D3 domains of CD4, respectively, but the two sites may exhibit allosteric interactions because the OKT4 mAb reversed the inhibitory activity of the RPA-T4 mAb (Fig. 3A, D). Competitive interactions of RPA-T4 and OKT4 were also noted at both 10 and 20 μg/mL concentrations (Fig. 3D). Given that anti-CD4 mAb are established inhibitors of MHCII-restricted antigen recognition but not IL-2 signaling, these data provide evidence that TCR recognition events were needed for continuous IL-2–dependent T cell expansion. The RPA-T4 mAb reliably inhibited growth in a bioassay of 5 d or longer but was not inhibitory in shorter 3-d assays (Fig. 3E). These data indicate that inhibition of TCR signaling requires time to impact IL-2 growth responses. Also, the RPA-T4 mAb exhibited optimal inhibition at higher cell densities (50,000 or 10^4.7^ cells/well), whereas the mAb lacked detectable inhibitory activity at lower cell densities (e.g. 12,500 or 10^4.1^ cells/well) (Fig. 3F). These data indicated that RPA-T4-mediated inhibition was optimal at cell densities favoring optimal cell clustering/contact. Overall, these data are consistent with a model whereby CD4 acts as a coreceptor to augment culture-intrinsic pMHCII/TCR interactions that elicit CD25^high^ expression and enable sustained growth in IL-2–supplemented cultures. The RPA-T4 mAb inhibited T cell growth by limiting IL-2 responsiveness rather than by limiting IL-2 bioavailability because IL-2 supplementation of cultures at subsequent phases of the bioassay did not reverse mAb-mediated inhibition (Fig. 3G). That is, the RPA-T4 mAb had similar inhibitory activity when IL-2 was added on days 0 and 2 (Fig. 3G, left two panels) or on days 0, 2, and 4 (Fig. 3G, right two panels) compared with a single IL-2 addition on day 0. These data were not consistent with the possibility that mAb-mediated inhibition was due to degradation or neutralization of a limited IL-2 pool. Rather, these data indicate that anti-CD4 mAbs blocked IL-2 responsiveness, perhaps by controlling CD25 expression.

**Figure 3:**
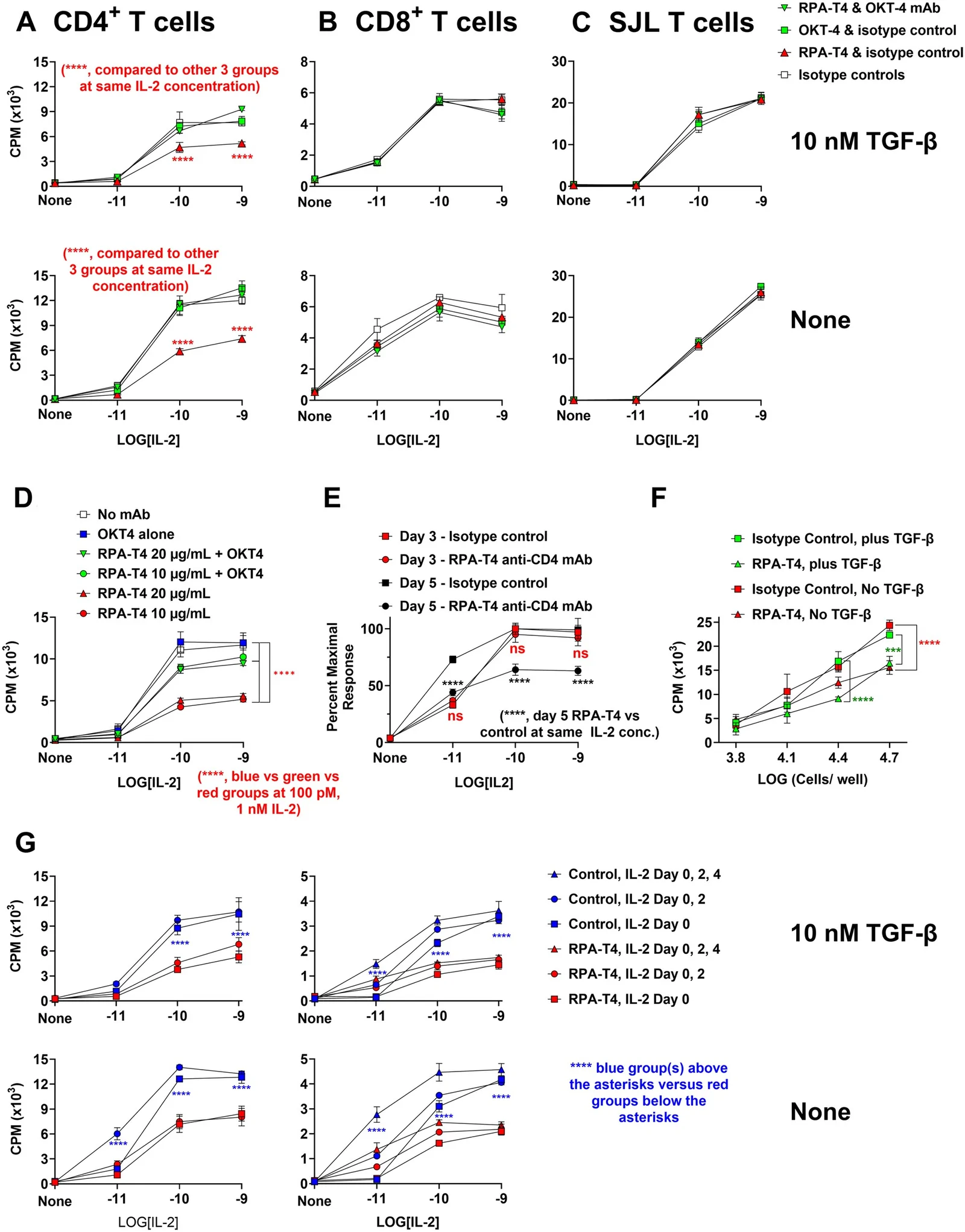
A neutralizing anti-CD4 mAb inhibited autologous MHCII-restricted autoantigen recognition and IL-2–dependent growth of CD4^±^ T-APCs. (A–G) Distinct CD4^+^ purified (A, D–G) and CD8^+^ purified (B) T-APC lines were initiated by a 3-d stimulation with PHA in the presence of 1 nM IL-2 and 10 nM TGF-β. Cells were passaged every 3 to 7 d in the presence of TGF-β and IL-2 for a total of 22 d (A), 80 d (B), 10 to 15 d (D-E), 78 d (F), 23 d (G, left two panels), or 36 d (G, right two panels). Human T cells (A, B, D–G) (50,000 cells/well, unless designated otherwise) or mouse SJL-PLP.1 T cells (C) (10,000 cells/well) were cultured with 10 pM, 100 pM, or 1 nM IL-2 (x-axis) in the presence or absence of anti-CD4 mAbs (OKT4 or RPA-T4) or isotype controls (MPC-11 or MOPC-21) in the presence or absence of 10 nM TGF-β. mAb concentrations were 20 μg/mL unless designated as 10 μg/mL. Cultures were pulsed with [^3^H]thymidine during the last 24 h of a 5-d or 6-d culture unless designated otherwise. T-APC lines used in these experiments were CL5 (A, D), P2 (B), murine SJL-PLP.1 (C), CL6 (E, F), or CL7 (G). Two-way analysis of variance with a Tukey’s multiple comparisons test was used to assess significance. ***P < 0.001, and ****P < 0.0001 for comparisons of the designated mean value(s) at the same IL-2 concentration. CPM, counts per minute; ns, not significant.

The FOXP3^+^ T cell lines used in these assays were derived via continuous expansion in IL-2 and TGF-β, and the bioassay was performed in the presence (Fig. 3A–C, G, top 3 panels) or absence of TGF-β (Fig. 3A–C, G, bottom 3 panels). Thus, cultures lacking TGF-β are T cell lines withdrawn from chronic TGF-β exposure. For CD4^+^ T cells, TGF-β was inhibitory in the bioassay (e.g. compare top and bottom panels of Fig. 3A, G). These data indicated that TGF-β enabled long-term growth competence while exerting short-term acute inhibition. The presence or absence of TGF-β did not alter RPA-T4 mAb-mediated patterns of inhibition.

### The anti-CD4 mAb RPA-T4 promoted a distinct CD25^low^ subset that had a diminished T-APC signature

Given the possibility that the RPA-T4 mAb may inhibit IL-2 responsiveness, we hypothesized that the RPA-T4 mAb would inhibit CD25 expression. Accordingly, long-term CD4^+^ T-APC cultures (53 d in culture) exhibited bimodal expression of CD25 (Fig. 4A, B), and a 15-d exposure to the RPA-T4 mAb decreased the CD25^high^ cell percentages from 72% to 34% (Fig. 4C, right). The CD25^high^ subset comprised larger blast cells compared with the CD25^low^ subset (Fig. 4C, left). Both CD25^high^ and CD25^low^ subsets expressed FOXP3, although the CD25^high^ subset expressed relatively higher levels of FOXP3 compared with the CD25^low^ subset (Fig. 4D). Notably, the RPA-T4 mAb primarily affected FOXP3 mean fluorescence intensity (MFI) values among CD25^high^ T-APCs (Fig. 4E, F). These data support the hypothesis that CD4-dependent TCR engagement of MHCII-restricted culture-intrinsic peptides is needed for autologous responsiveness, which is required for maintenance of a CD25^high^ phenotype and competitive IL-2–dependent growth.

**Figure 4:**
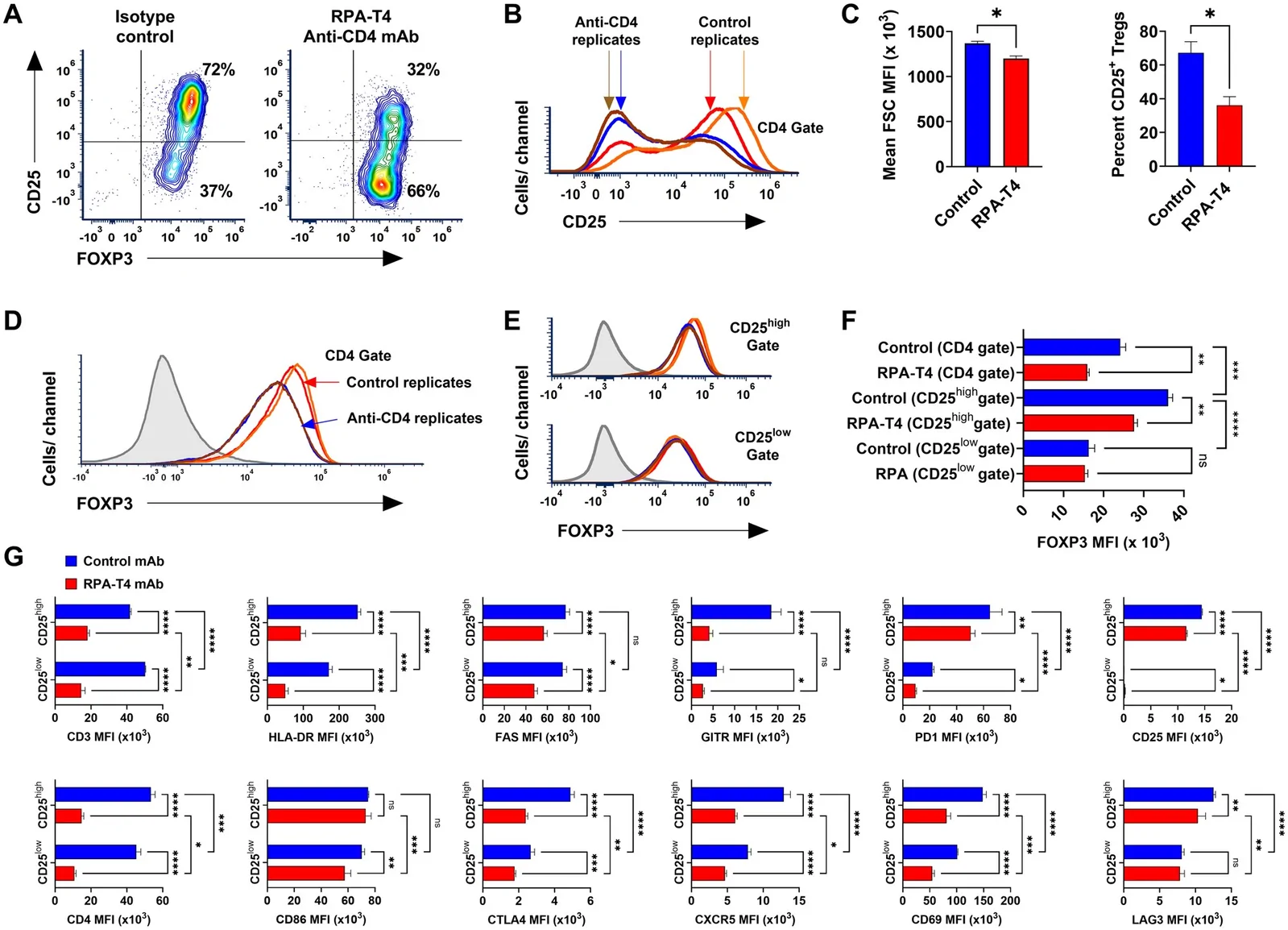
The anti-CD4 mAb RPA-T4 promoted a distinct CD25^low^ subset that had a diminished T-APC signature. A CD4^+^ purified T cell line was initiated by a 3-d stimulation with PHA in the presence of 1 nM IL-2 and 10 nM TGF-β. These T cells were passaged in the presence of TGF-β and IL-2 for a total of 53 d (A–F) or 100 d (G). During the last 2 wk, T cells were cultured in the presence of 20 μg/mL RPA-T4 mAb or the isotype control mAb, in the presence of 1 nM IL-2 and 10 nM TGF-β. Cells were stained with CD3 PerCP-Cy5.5 (HIT3a), CD4 BV421 (OKT4), CD8 AF700 (SK1), CD25 PE (M-A251), and FOXP3 AF488 (150D) (A–F), and/or antibodies against HLA-DR, DP, DQ SparkBlue574 (Tu39), CD86 BV650 (FUN-1), FAS BUV737 (DX2), CTLA4 AF647 (BN13), GITR BV711 (108-17), CXCR5 BV605 (MU5UBEE), PD1 PE-Cy5 (EH12.2H7), CD69 PE (FN50), CD25 APC-Fire750 (BC96), and LAG3 BUV563 (3DS223H). (A, B) Representative FOXP3 × CD25 dotplots are shown with the respective CD25 histograms. (C) Bar charts show mean forward scatter MFI values (left) and CD25^high^ percentages (right). (D–F) FOXP3 histograms of CD4-gated and CD25^high^/CD25^low^ gated subsets are shown for RPA-T4–treated versus control T cell cultures together with the respective FOXP3 MFI values. (G) Cultures treated with 20 μg/mL RPA-T4 or isotype control mAbs were also compared for other markers in CD25^high^ versus CD25^low^ subsets. CD4^+^ purified T cells from the P3 line (A–F) or the CL1 line (G) were used in these experiments. Analyses were 2-tailed unpaired *t* tests (C) or 2-way analysis of variance with Tukey’s multiple comparisons tests (F, G). *P < 0.05, **P < 0.01, ***P < 0.001, ****P < 0.0001. FSC, forward scatter; ns, not significant.

Previous studies showed that continued TCR recognition of positively selecting peptides in extrathymic tissues not only promoted mature T cell survival but also maintained mature T cell competence by enhancing reactivity to foreign antigens.^28^^,^^59–62^ To test whether self-peptide recognition was required to maintain reactive competence in this system, we assessed whether the RPA-T4 antagonist impacted proteins essential for T-APC activity or TCR reactivity (Fig. 4G). A 2-wk exposure to the anti-CD4 RPA-T4 mAb reduced expression of CD3 and CD4, which are required for antigen responsiveness, as well as HLA-DR and CD86, which mediate T cell antigen presentation and costimulation. These findings indicate that ongoing culture-intrinsic antigen recognition is required to maintain both antigen responsiveness and efficient T cell–mediated antigen presentation. The RPA-T4 mAb also inhibited expression of additional markers, including CD95 (FAS), CTLA4, GITR, CXCR5, PD1, CD69, and LAG3. Overall, these data indicate that ongoing TCR engagement maintains an autologous self-reinforcing cycle of T cell–mediated antigen presentation, TCR engagement, IL-2 responsiveness, and T-APC phenotypic identity.

### T-APC lines presented agonistic self-antigens and activated polyclonal Tcon responses while seeding new T-APC lines

Figures 3 and 4 supported the hypothesis that the T-APC phenotype was associated with self-stimulatory pMHC/TCR interactions. This hypothesis was supported by data showing that JL2.C5 T-APCs constitutively expressed CD40L, together with HLA-DR and CD25 (Fig. 5A). TCR-dependent activation is a primary instigator of CD40L expression with cytokines such as IL-2 capable of amplifying CD40L expression in activated T cells.^63–70^ These data thereby support the hypothesis that JL2.C5 T-APC TCRs may directly and/or indirectly sustain the CD40L-CD40-B7 costimulatory pathway. JL2.C5 expression of CD40L was inhibited by the anti-MHCII mAb L243, whereas the isotype control mAb had no inhibitory activity (Fig. 5B), and the anti-CD40L clone 5C8 blocked CD40L expression (Fig. 5C). The 5C8 and 24–31 anti-CD40L clones were used for neutralization and flow cytometric detection, respectively, because these noncompetitive anti-CD40L clones recognize separate epitopes.^71^ Thus, the inhibitory mechanism of the 5C8 clone may involve CD40L downregulation in addition to neutralization.

**Figure 5:**
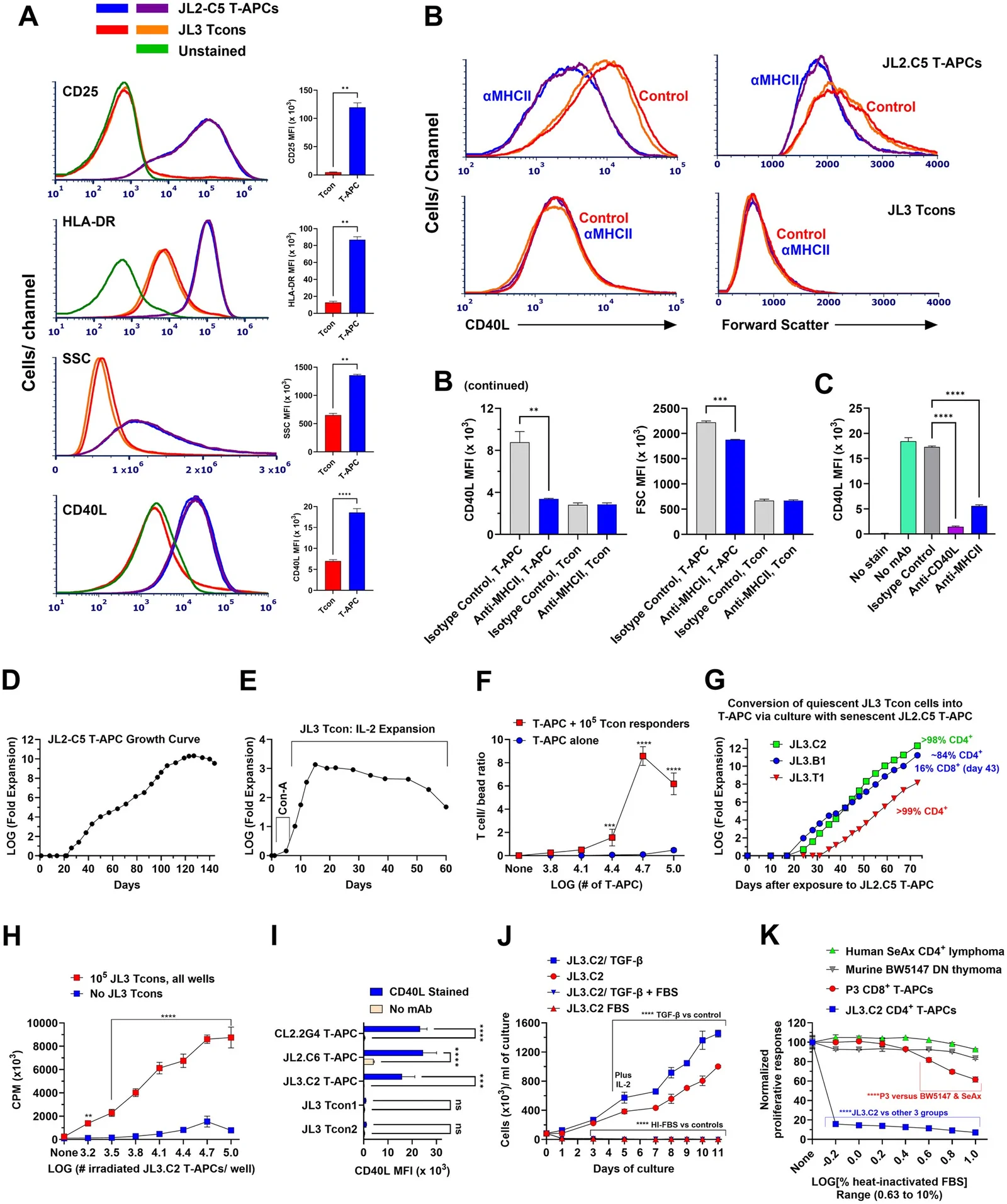
T-APC lines presented agonistic self-antigens and elicited polyclonal Tcon responses while seeding new T-APC lines. (A) JL3 Tcons were derived from freshly isolated PBMCs that were activated with Con-A and 1 nM IL-2 for 4 to 5 d. Tcons were passaged in 1 nM IL-2 in cRPMI media every 4 to 7 d for an additional 29 d. JL2.C5 T-APCs were derived in the presence of GM-CSF, IL-4, IL-2, and TGF-β (1 nM each). JL2.C5 cells were propagated in IL-2 and TGF-β for a total of 92 d (total expansion 10^7.4^-fold). Cells were stained with CD3 BV510 (SK7), CD4 BUV496 (SK3), CD8 Spark Blue 574 (SK1), CD25 PE-Fire 700 (MA251), HLA-DR Spark PLUS UV395 (L243), and CD40L PE (24-31). (B) JL2.C5 T-APCs (day 102) and JL3 Tcons (day 41 postactivation) were cultured for 24 h in the presence or absence of the anti-MHCII mAb L243 or the isotype control (10 μg/mL). (C) Similarly, JL2.C5 T-APCs (day 136) were cultured for 24 h in the presence or absence of anti-MHCII L243, anti-CD40L 5C8, or the isotype control (10 μg/mL). (D, E) Shown are the growth curves for the JL2.C5 T-APCs and the JL3 Tcon lines. (F) Designated densities (x-axis) of senescent nonexpanding JL2.C5 T-APCs (day 129) were added to 96-well plates with JL3 Tcons (day 16, 10^5^/well) in LymphoOne and 1 nM IL-2. Cells were passaged into 24-well plates on day 11. JL3 Tcon expansion was enumerated on day 18 with CountBright Absolute Counting Beads. (G) Cultures derived from panel F were expanded to T25 flasks, and after 2 to 4 wk 3 of these 18 cultures exhibited blastogenic T cell outgrowth. The initiation of the JL3 Tcon/JL2.C5 T-APC coculture (F) was designated as day 0 for initiation of the JL3.B1, JL3.C2, and JL3.T1 lines. (H) To assess whether the JL3.C2 T cells exhibited T-APC activity, JL3 Tcons (10^5^/well, day 39 out of activation) were cultured with designated densities (x-axis) of irradiated JL3.C2 T-APCs (5 Gy) for 5 d in the absence of exogenous mitogen or antigen. Cultures were pulsed with [^3^H]thymidine during the last 48 h of culture. (I) T-APC lines CL2.2G4 and JL2.C6 and JL3 Tcon lines were compared with the nascent JL3.C2 T-APC line (day 32) for CD40L expression. (J) JL3.C2 T-APCs were cultured without exogenous TGF-β through day 58 and then were cultured with or without 1 nM TGF-β for 4 d. Cells were then subjected to a 10-day passage in duplicate cultures with 1 nM IL-2 in the presence or absence of 1 nM TGF-β without and without 10% heat-inactivated FBS, with manual counts on days 1, 3, 5, and 7 to 11. Cultures were supplemented with 1 nM IL-2 on day 5. (K) CD4^+^ JL3.C2 (day 70), CD8^+^ P3 T-APCs (day 14), murine BW5147α/β^−^ cells, or SeAx cells were cultured in triplicate in a 2-d proliferative assay ([^3^H]thymidine pulse during the last 24 h) with 1 nM IL-2 in the presence of designated titrations of heat-inactivated FBS. (B, C, I) Data were assessed with 1-way analysis of variance with Sidak’s or Dunnett’s multiple comparison test, respectively. (F, H, J, K) Data were assessed with a 2-way analysis of variance with Sidak’s multiple comparison test. **P < 0.01, ***P < 0.001, ****P < 0.0001.

An important question was whether T-APC lines presented self-antigens to syngeneic Tcons. To address this question, we tested whether JL2.C5 T-APCs stimulated syngeneic responder JL3 Tcons. JL2.C5 T-APCs were derived from PBMCs and were propagated in 1 nM IL-2 and 1 nM TGF-β in xeno-free media. The JL2.C5 growth curve (Fig. 5D) shows that cultures were stationary for 2 to 3 wk before onset of exponential growth. These T-APCs then exhibited an approximate 10^10^-fold expansion over 3 to 4 mo before gradually becoming senescent wherein 100% of cells exhibited a nonproliferative, enlarged, granular CD8^+^ phenotype. Nonetheless, these T-APCs continued to express MHCII and costimulatory molecules. Conversely, JL3 Tcons were small, rested cells that did not express CD25 or CD40L and had limited expression of HLA-DR (Fig. 5A, B). These Tcons were PBMCs that were initially activated with the mitogen Con-A and 1 nM IL-2 in cRPMI (with 10% fetal bovine serum [FBS]) for 3 to 5 d. Tcons were then propagated at 1 nM IL-2 without mitogen with passages every 4 to 7 d until these cells became rested quiescent lines. The JL3 Tcon growth curve is shown in Figure 5E. After Con-A activation, JL3 blast cells expanded over 7 to 10 d but gradually downregulated CD25 and by 2 wk postactivation became small quiescent cells (Fig. 5A, E). During the “rest” phase of culture, Tcons were maintained in 1 nM IL-2 which enabled survival but not proliferation. Con-A activation caused differentiation of naïve Tcons into effector and memory T cells. However, because effector T cells have finite lifespans, rested JL3 Tcons represent long-lived quiescent memory T cells.

To assess syngeneic T-APC/responder interactions, senescent JL2.C5 T-APCs were cocultured with rested JL3 Tcons (11 d postactivation) in LymphoOne media and 1 nM IL-2 (Fig. 5F). In these assays, large, granular, and nondividing T-APCs and small, nondividing, nongranular Tcons were readily distinguishable. The T-APC/Tcon coculture resulted in the uniform blastogenesis and expansion of Tcons as assessed by use of CountBright Absolute Counting Beads. The presence of T-APCs was required for Tcon growth, as cultures initiated with Tcons but no T-APCs showed no Tcon activation. In cultures initiated with T-APCs but no Tcons, senescent T-APCs were stationary and lacked any evident growth. In the presence of T-APCs, expanding Tcons were passaged to 24 well cultures and then to T25 flasks. Of the 18 cultures seeded in 24-well plates (3 cultures at each T-APC density, 6 different T-APC densities = 18 cultures), 3 cultures exhibited de novo emergence and sustained exponential growth of new T-APC lines including JL3.C2, JL3.T1 (both >98% CD4^+^) and JL3.B1 (∼85% CD4^+^) by day 51 (Fig. 5G). Because the JL2.C5 T-APCs were CD8^+^ and senescent, we conclude that emergent CD4^+^ T-APCs were derived from memory Tcons. Day 0 represents the day Tcons were mixed with JL2.C5 T-APCs. Like T-APC lines sourced directly from PBMCs, lines derived from Tcons exhibited a ∼2-wk incubation phase, then showed exponential expansion with near 100% viability with no evidence of exhaustion. To confirm that Tcon-derived T-APCs had T-APC activity, JL3.C2 T-APCs were irradiated (5 Gray) and cultured with syngeneic rested JL3 Tcons in the absence of exogeneous antigens or mitogens (Fig. 5H). These experiments showed that the JL3.C2 T-APCs stimulated proliferation of syngeneic Tcons whereas control cultures comprised of Tcons alone, or irradiated JL3.C2 T cells alone, lacked proliferative activity. Constitutive CD40L expression by JL3.C2 blast cells provided additional evidence that these T cells had a T-APC phenotype (Fig. 5I). Unlike T-APC lines derived from freshly isolated PBMCs, Tcon-derived T-APCs initially did not require an exogenous source of TGF-β during the first 70 d of expansion. Nonetheless, JL3.C2 T-APCs responded to TGF-β by exhibiting enhanced T cell expansion (Fig. 5J).

During our initial experiments, we subjectively noted PBMC-derived T-APCs required a xeno-free culture system to initiate and sustain spontaneous growth. To assess whether Tcon-derived JL3.C2 T-APCs also had the same requirement, we tested whether JL3.C2 T cells were negatively impacted by heat-inactivated FBS, which represents a complex foreign antigen in an otherwise xeno-free system. These experiments showed that these T-APCs did not expand in the presence of 10% FBS (Fig. 5J) and that CD4^+^ T-APCs were substantially more sensitive than CD8^+^ T-APCs to the negative action of FBS (Fig. 5K). This differential sensitivity may reflect greater entry of foreign peptides into the exogenous MHCII antigen processing pathway compared with the endogenous MHCI pathway. Conversely, FBS had no inhibitory action on control murine BW5147 thymoma cells or human SeAx T cells. These data support the conclusion that Tcon-derived T-APCs, like PBMC-derived T-APCs, require a xeno-free system to support spontaneous growth, which is predicted to be a cardinal generalized feature of the T-APC subset. These data indicate that xeno-free conditions represent a requirement, rather than just a feature, of T-APC culture systems.

### Tcon-derived JL3.C2 T-APCs expressed a professional APC phenotype

We next asked whether Tcon-derived T-APC lines expressed a professional APC phenotype. Nascent Tcon-derived JL3.C2 and JL3.B1 T-APC lines were compared with established PBMC-derived JL2.C5 and JL2.C6 T-APC lines and to the parental JL3 Tcon6 line (Fig. 6A, B). Tcon-derived T-APCs (day 39) and PBMC-derived T-APCs (day 32) had similar expansion lifetimes. Statistical analyses focused on CD4^+^ cells, which were the main drivers of these nascent T-APC lines. CD4^+^ JL3.C2 and the CD4^+^ subset of JL3.B1 had T-APC phenotypes comparable to the established T-APC lines based on high level expression of HLA-DR, CD80, CD86, and surface CD74 (class II–associated invariant chain) along with high-level expression of CD25 and CD134 (OX40) (Fig. 6B). Because these T-APCs also expressed high levels of CD28, one would presume that autologous costimulatory pathways were operational. Notably, both PBMC-derived and Tcon-derived T-APCs also abundantly expressed the CD123/IL-3Rα subunit, which is a prominent marker expressed on plasmacytoid DCs.^72^ The JL3.B1 CD4^+^ subset (∼25%) had higher levels of APC/activation markers compared with the JL3.B1 CD8^+^ subset. In contrast, the established (parental) JL2.C5 T-APC line was ∼80% CD8^+^ single positive at this juncture in the time course, and both CD4^+^ and CD8^+^ subsets had a pronounced T-APC phenotype comparable to the CD4^+^ T-APC lines. Subsequently, the JL3.B1 line evolved such that the CD4^+^ single-positive subset became predominant by day 51. In contrast to the nascent and established parental T-APC lines, the JL3 Tcon6 subline lacked T-APC/activation markers. Overall, these data show that the established senescent JL2.C5 T-APC line (Fig. 5D) elicited a new life cycle of activated, exponentially expanding CD4^+^ T-APC lines from nonactivated, quiescent memory Tcons. Thus, T-APCs elicited memory T cell differentiation into new T-APC lines.

**Figure 6:**
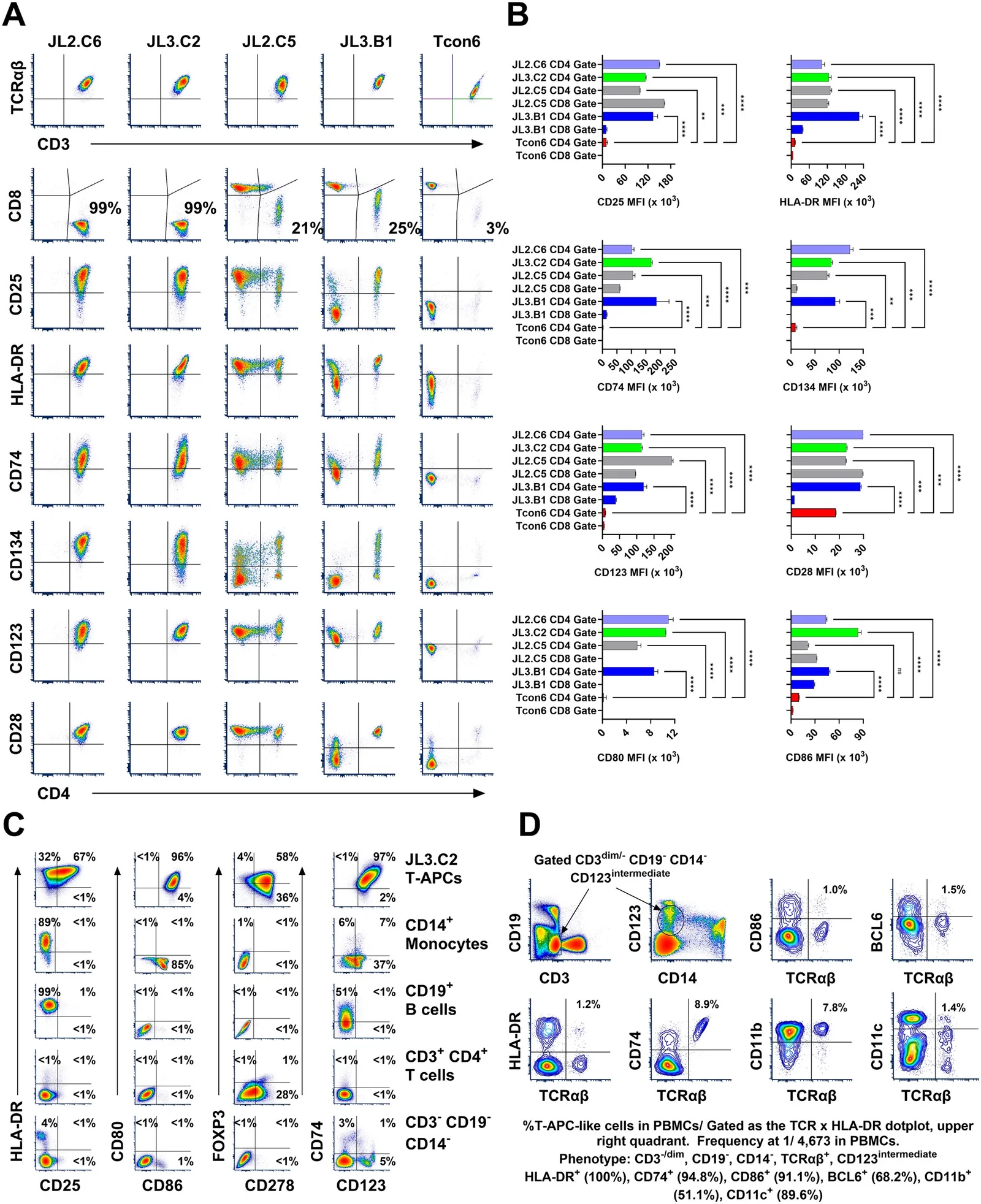
Tcon-derived JL3.C2 T-APCs expressed a professional APC phenotype. (A, B) The Tcon-derived JL3.C2 and JL3.B1 T-APC lines were compared with established T-APC lines (JL2.C5 and JL2.C6) and to the parent Tcon line (JL3 Tcon6). (A) Cells were stained with TCRα/β PE/Cy7 (IP26), CD3 BV785 (SK7), CD4 BV421 (OKT4), CD8 Alexa Fluor 700 (SK1), CD28 BV605 (CD28.2), CD25 FITC (M-A251), CD74 PE (LN2), CD80 BV711 (2D10), CD86 BV650 (FUN-1), CD123 PerCP/Fire806 (S18016C), OX40/CD134 PE-Cy5 (ACT35), and HLA-DR Spark PLUS UV395 (L243). (B) Mean MFI values are shown for CD4 and CD8 gated cell lines. Statistical analyses were restricted to CD4 gated values and were assessed by 1-way analysis of variance with Tukey’s multiple comparison test. **P < 0.01, ***P < 0.001, ****P < 0.0001. (C, D) JL3.C2 T-APCs and PBMCs (day 0, no culture) were stained with TCRα/β PE/Cy7 (IP26), CD3 BV510 (SK7), CD4 BV421 (OKT4), CD8 Spark Blue 574 (SK1), CD11b StarBright UV575 (M1/70), CD11c BV785 (Bu15), CD14 PE/Fire 700 (S18004B), CD19 StarBright UV795 (HIB19), CD25 APC/Fire 750 (BC96), CD74 PE (LN2), CD80 BV711 (2D10), CD86 BV650 (IT2.2), CD278 BV605 (C398.4A), and HLA-DR Spark Plus UV395 (L243). The same flow gain settings were used for comparison of JL2.C2 T-APCs and PBMCs. ns, not significant.

To validate that T-APCs had a hybrid phenotype combining canonical features of both professional APCs and regulatory T cells, JL3.C2 T-APCs were directly compared with relevant cell types in peripheral blood (Fig. 6C). JL3.C2 T cells expressed professional APC markers (HLA-DR, CD74, CD80, CD86, and CD123) and canonical Treg/T follicular regulatory cell (Tfr) markers (CD25, FOXP3, ICOS) at levels matching or exceeding those on peripheral B cells, monocytes, or Tregs. To understand whether the T-APC phenotype was generated de novo during in vitro culture or whether T-APCs have an in vivo counterpart, we assayed peripheral blood for T-APC–like cell types (Fig. 6D). These studies revealed a rare TCRα/β CD3^dim/−^CD123^intermediate^ CD19^−^ CD14^−^ population that was HLA-DR^+^. A high frequency of gated TCRα/β HLA-DR^+^ T cells also expressed CD74 (95%), CD86 (91%), CD11c (90%), BCL6 (68%), and CD11b (51%). This T-APC–like subset had a frequency of less than 0.025% of blood PBMCs. Future research will be required to know whether this subset represents a T-APC precursor. The TCRα/β CD3^dim/−^ phenotype was paradoxical due to the obligate coexpression of TCRα/β and CD3ε on the T cell surface. Thus, we presume that CD3ε is present but that the SK7 CD3ε epitope is masked by other surface molecules in this subset. Overall, these data provide evidence that T-APCs have robust APC and regulatory phenotypes and that rare stem-like T-APC precursors may circulate in blood.

### IL-2 and TGF-β are required for respective short- and long-term viability of continuous T-APC lines

Because T-APCs appeared to include defining features of both professional APCs and FOXP3^+^ Tregs, cytokine withdrawal experiments were used to assess requirements for the canonical Treg-associated cytokines IL-2 and TGF-β. A JL3.C2 T-APC subline was used in which parallel cultures were propagated from day 39 to day 78 with or without 1 nM TGF-β. As shown in Figure 7A to C, IL-2 withdrawal resulted in robust reductions of CD25 and CD86 by day 3 followed by extensive death by day 6. HLA-DR, FOXP3, and CD95 (FAS) were also downregulated following IL-2 withdrawal. In contrast, IL-2 withdrawal caused enhanced CD3 expression. IL-2 withdrawal resulted in a modest downregulation of FOXP3 by day 1 but with no further FOXP3 loss on days 3 and 6. These results are consistent with the hypotheses that IL-2 was necessary to sustain CD25 on CD25^high^ T-APCs and that robust IL-2 signaling was needed to maintain T-APC viability.

**Figure 7:**
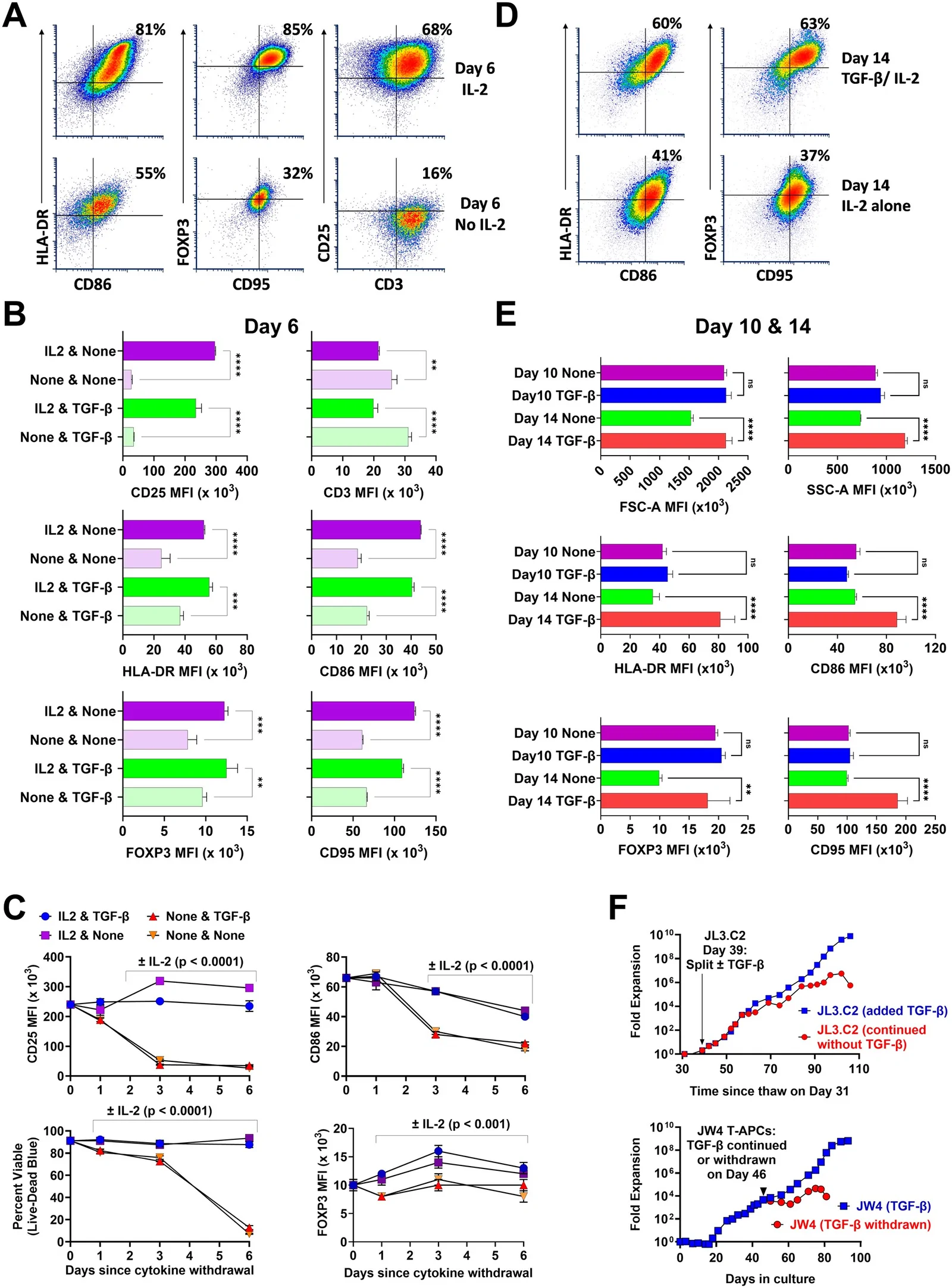
IL-2 and TGF-β are required for respective short- and long-term viability of continuous T-APC lines. A JL3.C2 T-APC subline was thawed on day 31, and parallel cultures were initiated on day 39 with or without 1 nM TGF-β. On day 78, the effects of IL-2 and/or TGF-β withdrawal were assessed via a time course experiment. (A–E) JL3.C2 TGF-β–cultured T-APCs were cultured with or without IL-2 in the presence or absence of 1 nM TGF-β from days 0 to 14 (n = 3). Passages were performed on days 3, 6, and 10. At each time point, cells were stained with CD3 BV510 (SK7), CD4 BV421 (OKT4), CD25 PE-Fire 700 (M-A251), CD86 BV650 (FUN-1), FAS PerCP-Fire806 (DX2), HLA-DR Spark Plus UV396 (L243), and FOXP3 Alexa Fluor 488 (150D). Dotplots (A, D) show representative positive control cultures (with IL-2 and TGF-β) in the top rows versus cytokine-deprived cultures in the bottom rows (CD3^+^ CD4^+^ gate). Quadrants depict reference comparators for positive controls versus cytokine-deprived groups, whereas all populations were positive compared with unstained negative controls. (B) IL-2 withdrawal for all groups was assessed on day 6 via a 1-way analysis of variance with Sidak’s multiple comparison test. (C) Mean values on days 1, 3, and 6 were compared by 2-way analysis of variance with a Tukey’s multiple comparison test. (E) TGF-β withdrawal was assessed for the remaining IL-2–supplemented groups on days 10 and 14 with a 1-way analysis of variance with Sidak’s multiple comparison test. (F) Shown are growth curves for JL3.C2 T-APCs and JW4 T-APCs that were cultured with and without TGF-β. **P < 0.01, ***P < 0.001, ****P < 0.0001. ns, not significant.

The consequences of TGF-β withdrawal were first evident on day 14 (Fig. 7D, E), as noted by loss of HLA-DR, CD86, FOXP3, and CD95 (FAS). Thus, the negative effects of TGF-β withdrawal were only realized during longer cultures. The presence or absence of TGF-β also determined long-term viability when cultivated past day 80 in that JL3.C2 T-APCs cultured without TGF-β died by day 106, whereas the TGF-β–supplemented culture maintained robust expansion. Likewise, the JW4 T-APC line also showed long-term dependence on TGF-β in that control JW4 T-APCs cultured without TGF-β faltered and died by day 81, whereas parallel cultures of JW4 T-APCs cultured with TGF-β maintained robust expansion. These data lend credence to the hypothesis that TGF-β is needed to maintain viability of CD25^high^ T-APCs during sustained exponential expansion.

### T-APCs encompassed distinct subsets marked by differential expression of CD25, CD69, and PD1

Given that IL-2 and TGF-β are critical for sustained viability and expansion of T-APCs, we next assessed cytokine concentration-response profiles. A TGF-β–conditioned CD4^+^ T-APC line (73 d in culture) was cultured in triplicate for 7 d in the presence of designated IL-2 concentrations (100 pM versus 1, 10, or 100 nM) in the presence or absence of 10 nM TGF-β. Although higher IL-2 concentrations are classically associated with higher CD25 expression, these experiments revealed a paradox in that higher IL-2 concentrations, in synergy with TGF-β, facilitated emergence of a CD25^low^ subset that coexpressed a CD69^high^ phenotype (Fig. 8A, B) and a PD1^high^ phenotype (Fig. 8C, D). In contrast, T-APCs maintained in low IL-2 concentrations were dominated by the canonical CD25^high^ CD69^low^ PD1^low^ phenotype together with only limited percentages of T-APCs expressing either or both CD69 and PD1 (Fig. 8A, C). The transition from a dominant CD25^high^ CD69^low^ PD1^low^ phenotype to a CD25^low^ CD69^+^ or CD69^+^ PD1^high^ phenotype is shown as a function of IL-2 and TGF-β concentrations (Fig. 8B, D).

**Figure 8:**
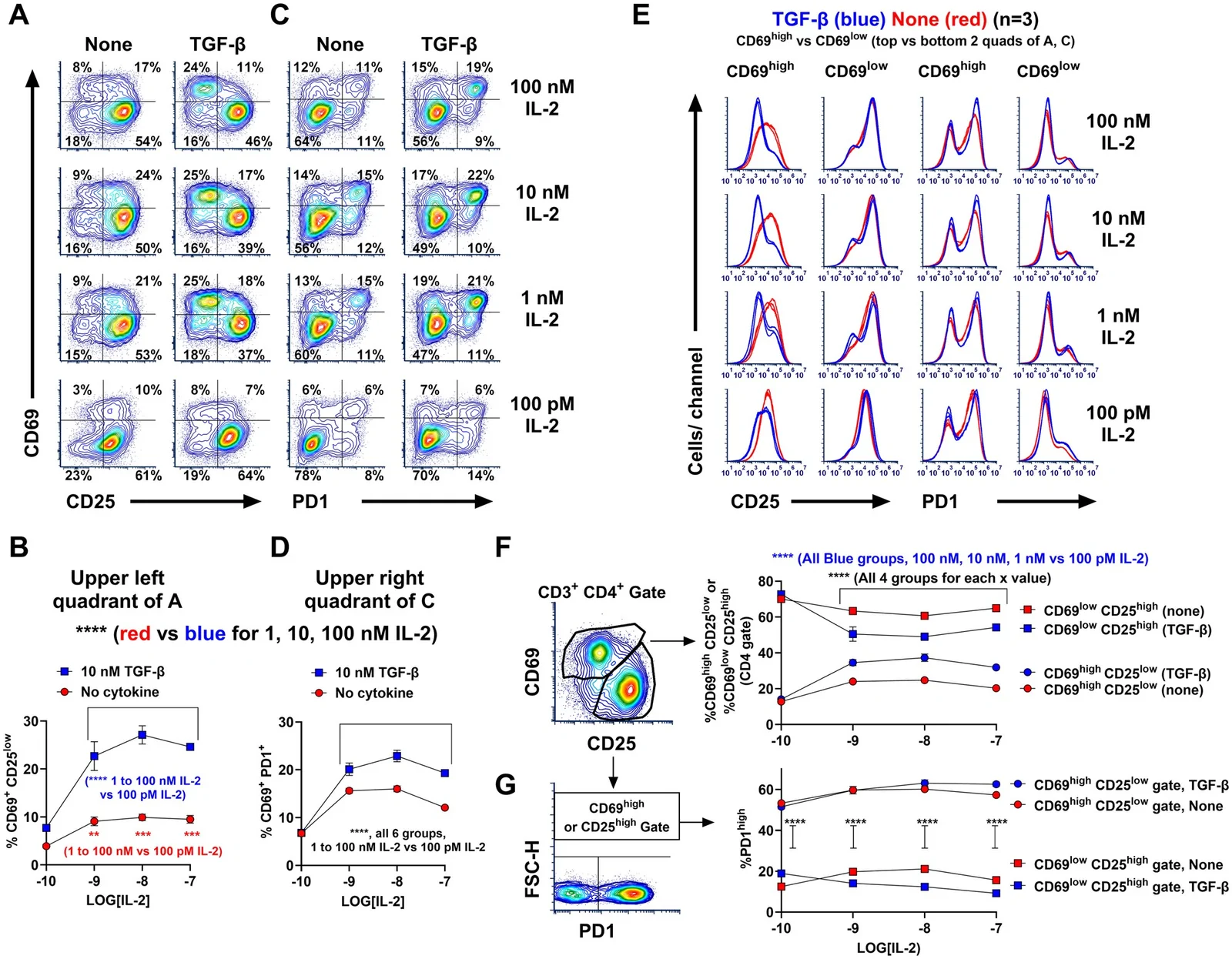
T-APCs encompassed distinct subsets marked by differential expression of CD25, CD69, and PD1. CD4^+^ T cells were purified from PBMCs and were activated for 3 d with PHA and IL-2 in the presence of 10 nM TGF-β. TGF-β–cultured T cells were then passaged every 3 to 7 d in IL-2 with TGF-β for 73 d. Cells were washed and cultured in triplicate at 500,000 cells/well in 24-well plates with 100 pM, 1 nM, 10 nM, or 100 nM IL-2 (x-axis) in the presence or absence of 10 nM TGF-β for 7 d. Cells were stained with CD3 NovaFluor Blue 610-70S (SK7), CD4 BV421 (OKT4), CD8 AF700 (SK1), CD25 APC-Fire750 (BC96), CD69 PE (FN50), and PD1 PE-Cy5 (EH12.2H7). These T cells were >99% CD4^+^ and lacked detectable CD8^+^ T cells. (A, B) CD3^+^ CD4^+^ gated T cells were analyzed in CD25 × CD69 dotplots (A), and the respective frequencies of CD25^low^ CD69^high^ T cells (upper left quadrant) are portrayed in the associated line graph (B). (C, D) CD3^+^ CD4^+^–gated T cells were analyzed in PD1 × CD69 dotplots (C), and the respective frequencies of PD1^high^ CD69^high^ T cells (upper right quadrant) are portrayed in the associated line graph (D). (E) Histograms of the combined top two quadrants or the combined bottom two quadrants from panels A and C show bimodality of CD25 and PD1 expression as a function of IL-2/TGF-β concentrations and CD69 expression. (F, G) After the 7-d culture with 100 nM IL-2 and 10 nM TGF-β, percentages of CD25^low^ CD69^high^ versus CD25^high^ CD69^low^ subsets (F) and percentages of PD1^high^ subsets (G) are shown in the respective right-hand line graphs. T-APCs from the CL6 line were used in these experiments. (B, D, F, G) A 2-way analysis of variance with a Tukey’s multiple comparisons test was used to assess differences between groups. **P < 0.01, ***P < 0.001, ****P < 0.0001. FSC-H, forward scatter height.

High concentrations of IL-2 and TGF-β promoted bimodality of CD25^low^ versus CD25^high^ subsets in both CD69^high^ and CD69^low^ gates (compare the first and second columns of Fig. 8E). Conversely, high concentrations of IL-2 and TGF-β did not qualitatively alter the bimodality of the PD1^high^/PD1^low^ phenotypes within each respective CD69^high^ and CD69^low^ subset, although the distribution of the PD1^high^/PD1^low^ phenotypes differed fundamentally between the CD69^high^ and CD69^low^ subsets (compare the third and fourth columns of Fig. 8E). These findings are portrayed graphically in Figure 8F, wherein TGF-β and IL-2 caused increased frequencies of CD69^high^ CD25^low^ T-APCs and a reciprocal loss of the opposite subset. The PD1^high^ phenotype was enriched in the CD69^high^ subset across a wide range of IL-2 and TGF-β concentrations (Fig. 8G). These data reveal 2 T-APC subsets, including a classical CD25^high^ subset as well as a distinct CD25^low^ CD69^high^ subset enriched for a PD1^+^ subpopulation. These analyses did not distinguish whether high IL-2/TGF-β concentrations elicited T cell differentiation or outgrowth of pre-existing subsets. One possibility is that high IL-2 concentrations saturated both low-affinity IL2Rβγ and high-affinity IL2Rαβγ receptors, thereby erasing the competitive advantage conferred by CD25. Overall, the CD25^high^ FOXP3^+^ population, which was dominant at low IL-2 concentrations, bifurcated into qualitatively distinct CD25^low^ and CD25^high^ populations when the culture was transitioned to high TGF-β/IL-2 concentrations.

### High concentrations of IL-2 and TGF-β caused dominance of CD25^low^ T-APCs even though CD25^high^ T-APCs maintained an enhanced IL-2 signaling signature

The CD25^high^ CD69^low^ PD1^low^ and CD25^low^ CD69^+^ PD1^+^ subsets had altered expression of other markers, which were analyzed at designated IL-2 concentrations (10 pM to 10 nM) with or without continued culture in 10 nM TGF-β (Fig. 9). These lines were cultured long term with TGF-β since initiation of the line and then were cultured for 7 d with or without TGF-β in the assay. These data presentations (Fig. 9A–T) required different y-axis scales to appropriately show relative differences. Many markers exhibited high fluorescence (MFI values >10^4^), including CD25, FOXP3, HLA-DR, CD86, CD3, CD4, CD95 (FAS), and CD69. Other markers exhibited lower levels of fluorescence (MFI values* <*10^4^), including CTLA4, CD80, CD28, CD127, PDL2, FASL, LAG3, GARP, and GITR.

**Figure 9:**
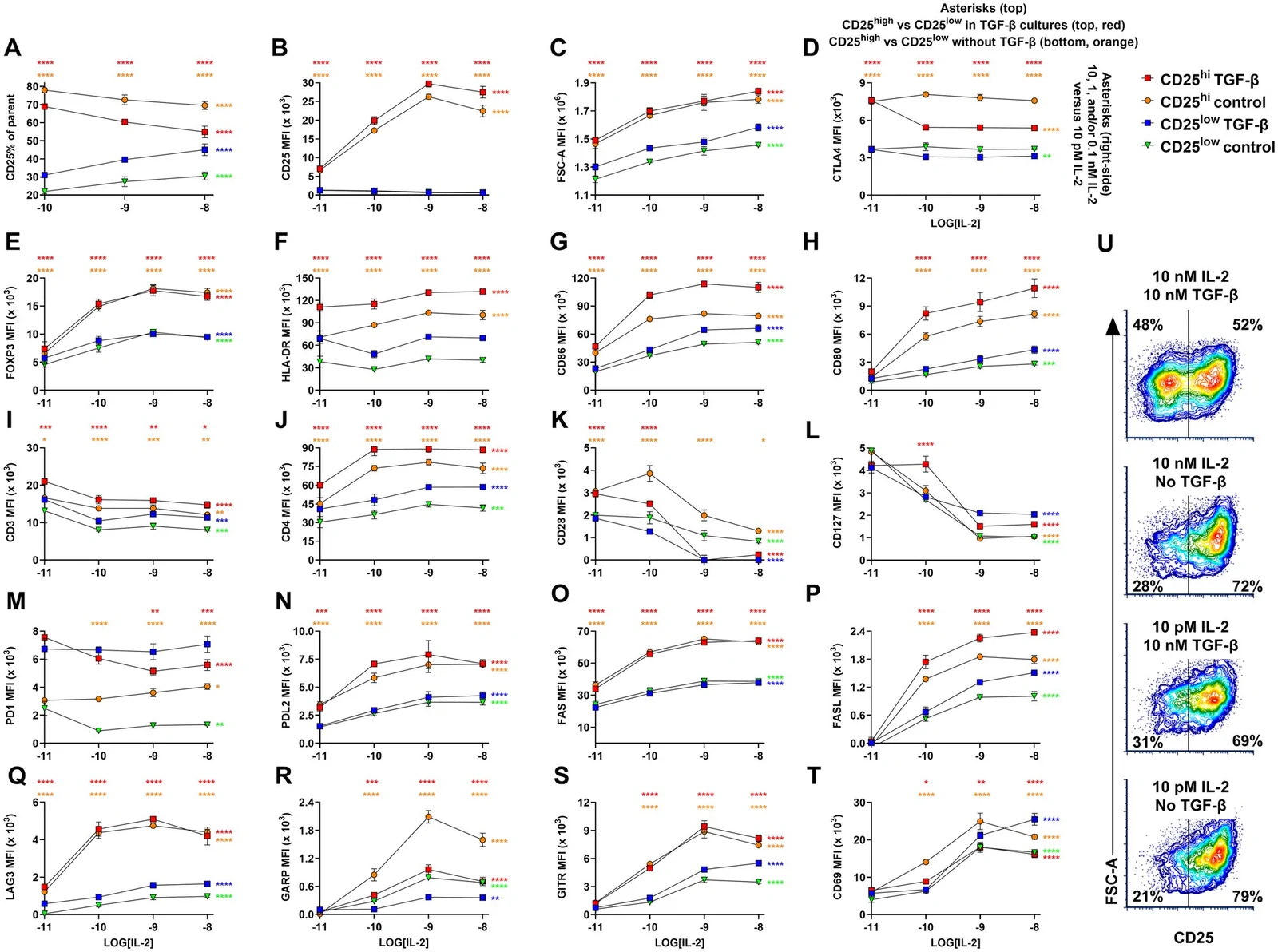
High concentrations of IL-2 and TGF-β caused dominance of CD25^low^ T-APCs even though CD25^high^ T-APCs maintained an enhanced IL-2 signaling signature. CD4^+^ T cells were purified from PBMCs and were activated for 3 d with PHA and IL-2 in the presence of 10 nM TGF-β. Cells were then passaged every 3 to 7 d in IL-2 and TGF-β for a total of 53 d. T cells were washed and cultured in triplicate at 500,000 cells/well in 24-well plates with 10 pM, 100 pM, 1 nM, and 10 nM IL-2 in the presence or absence of 10 nM TGF-β for 7 d. Cells were stained (via multiple panels) with CD3 NovaFluor Blue-610 (SK7), CD4 BV421 (OKT4), CD8 AF700 (SK1), CD25 APC-Fire750 (BC96), CD25 PE (MA251), CD28 BV510 (CD28.2), CD69 PE (FN50), CD80 B711 (2D10), CD86 BV650 (FUN-1), CD95/FAS BUV615 (DX2), CD127 PE-Cy5 (A019D5), CTLA4 AF647 (BN13), CD178/FASL BUV737 (NOK-1), CD273/PDL2 PE-Cy7 (MIH18), CD279/PD1 PE-Cy5 (EH12.2H7), GITR BV711 (108-17), FOXP3 AF488 (PCH101), HLA-DR BUV395 (G46-6), GARP PE-Cy7 (7B11), and LAG-3 BUV563 (3DS223H). T cells were >99% CD4^+^ and lacked detectable CD8^+^ T cells. (A) Percentages of CD25^high^ and CD25^low^ subsets and the respective MFI values of markers (B–T) are portrayed as a function of IL-2 concentration. Note the use of different y-axis scales. (U) CD3^+^ CD4^+^ gated T cells were analyzed in CD25 × forward scatter area (FSC-A) plots to distinguish CD25^low^ versus CD25^high^ subsets. T cells from the CL7 line were used in these experiments. A 2-way analysis of variance with a Tukey’s multiple comparisons test was used to assess differences between groups. *P < 0.05, **P < 0.01, ***P < 0.001, ****P < 0.0001. FSC-A, forward scatter area.

An interesting finding was that the CD25^high^ subset had superior IL-2 response signatures, but paradoxically the CD25^low^ subset had a growth advantage at high IL-2 concentrations, as shown by increased CD25^low^ versus CD25^high^ percentages (Figs. 8 and 9A, U). CD25^high^ T-APCs had superior IL-2–dependent response signatures compared with CD25^low^ subsets including increased cell size (forward scatter) and increased expression of CD25, FOXP3, HLA-DR, CD86, CD80, CD4, PDL2, CD95 (FAS), FASL, LAG3, GARP, and GITR. Conversely, CD25^low^ T-APCs exhibited superior expansion at higher IL-2 concentrations. A notable exception was that TGF-β, rather than IL-2, was pivotal for eliciting increased PD1 expression (Fig. 9M). In the CD25^high^ subset, TGF-β increased PD1 expression at low IL-2 concentrations, whereas in the CD25^low^ subset, TGF-β increased PD1 expression across a broad range of IL-2 concentrations. Although PD1 is known as an exhaustion marker, the same PD1 pathway serves as a negative regulatory buffer that protects T cells from overstimulation by mitigating excessive TCR and cytokine signaling intensities, which thereby preempts T cell exhaustion. These data are consistent with the hypothesis that a TGF-β/PD1 axis may mitigate excessive TCR and cytokine signaling to preserve exponential T-APC expansion. Due to higher PD1 expression levels, the TGF-β/PD1 axis may mediate more prominent protection in the CD25^low^ T-APC subset may thereby account for superior CD25^low^ T-APC growth at high IL-2 concentrations.

In comparing two of our T-APC lines, PD1 expression in the CL6 T cell line (Fig. 8) was substantially higher than PD1 expression levels in the CL7 line (Fig. 9) (PD1 MFI values > 10^5^ compared with PD1 MFI values* <*10^4^, respectively). These data suggested that PD1-mediated regulation may vary substantially among different T-APC culture systems, particularly during evolution of these lines. Compared with CD25^low^ T cells, CD25^high^ T cells expressed higher levels of PDL2 by an IL-2–driven mechanism, although expression levels were relatively low (<10^4^ MFI values) (Fig. 9N). Differential MFI values obtained for PD1 versus PDL2 detection likely represented different expression levels, because both PD1 and PDL2 mAbs were coupled with bright fluorochromes (PE-Cy5 and PE-Cy7, respectively). PDL1 was not detected on these T cell subsets (APC-eFluor780, MIH1). Thus, TGF-β–dependent PD1 signaling pathways were present in these T-APC cultures and were well positioned as regulators of these subsets.

Both CD25^high^ and CD25^low^ subsets exhibited IL-2–dependent downregulation of CD28, and this inhibitory mechanism was potentiated by TGF-β (Fig. 9K). Both subsets also showed an IL-2–dependent inhibition of CD127 (IL7Rα) expression (Fig. 9L), which was expected given that IL-2–activated T cells exhibit a CD127^low^ phenotype.

### T-APC coculture with syngeneic polyclonal Tcons caused an initial activation followed by fratricidal collapse

Given their robust APC activity and prominent regulatory systems (e.g. PD1, CD95), T-APCs may mediate important regulatory functions. Previous coculture systems utilized senescent or irradiated T-APCs to avoid the overwhelming exponential T-APC growth in T-APC/responder assays (Fig. 5F, H). Because senescence or irradiation may limit T-APC activity, we also tested exponentially expanding JL3.C2 T cells in T-APC/responder assays (Fig. 10). The CD4^+^ JL3.C2 T-APC line and JL3 Tcons were cultured alone or together with 1 nM IL-2 at a 1:10 ratio (10^4^ T-APCs + 10^5^ Tcons/well) in the absence of exogenous antigens or mitogens. Cultures were subjected to flow analysis on day 9. T-APCs and Tcons were distinguished by lineage markers in that T-APCs were uniformly CD4^+^, and Tcons were 88% CD8^+^, 9% DN, and 2% CD4^+^ (Fig. 10A). Thus, any phenotypic difference in the CD8^+^ or DN Tcon subset with or without CD4^+^ T-APC coculture would reflect an altered activation profile of Tcon responders. Statistical analyses focused on the primary question of whether the T-APC/Tcon cocultures caused DN or CD8^+^ Tcon activation compared with Tcons cultured alone. T-APCs uniformly elicited broad activation of Tcon responders which acquired high expression of APC/activation markers HLA-DR, CD74, CD25, CD134, CD28, and CD86 on both CD8^+^ or DN Tcon subsets (Fig. 10A, B). These data support the conclusion that CD4^+^ T-APCs conferred an activated T-APC phenotype on CD8^+^ or DN memory Tcons in the absence of mitogen or exogenous antigen.

**Figure 10:**
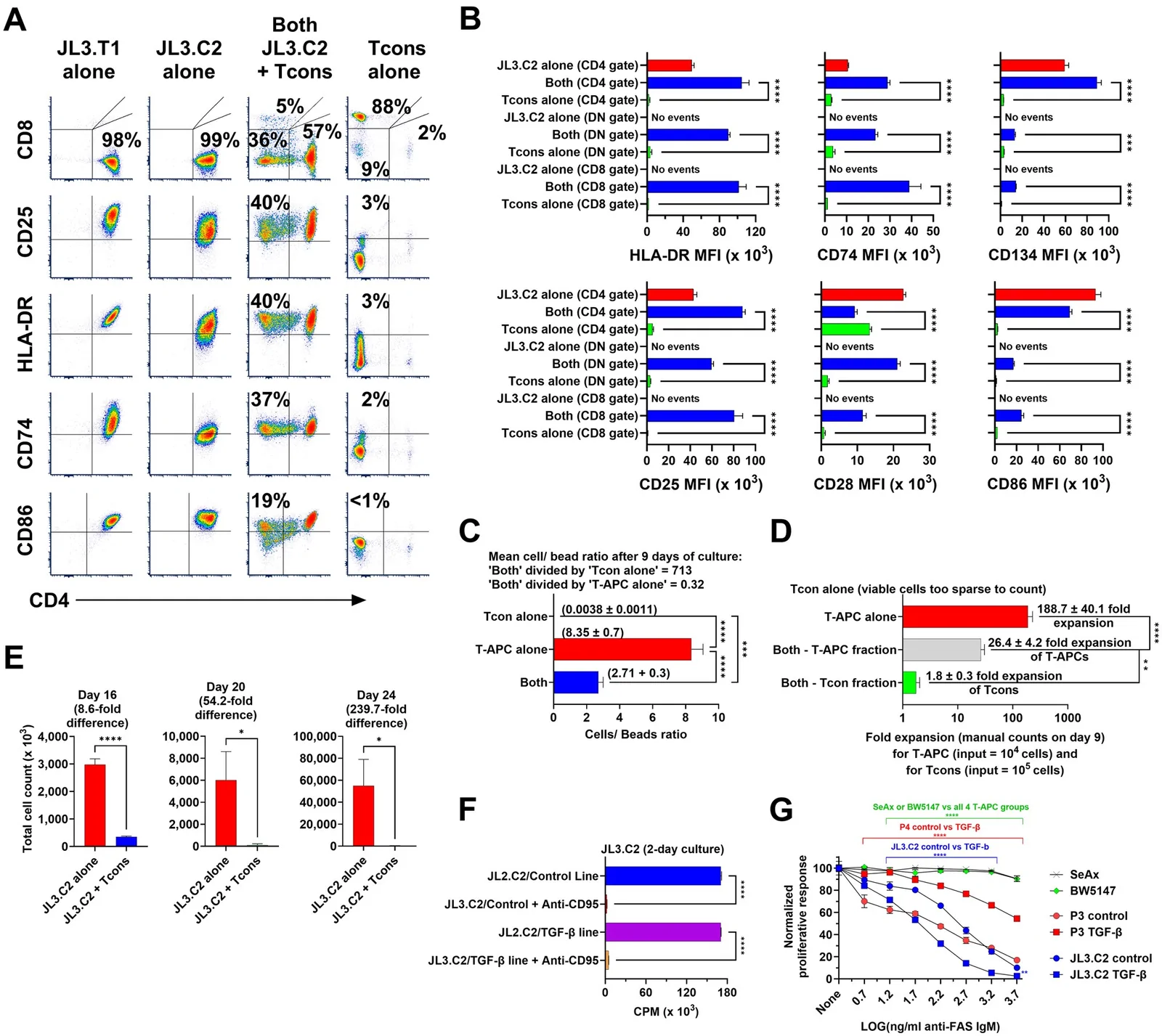
T-APC coculture with syngeneic polyclonal Tcons caused an initial activation followed by fratricidal collapse. (A–D) The JL3.C2 T-APC line (day 43, 10^4^/well) and JL3 Tcons (day 39 of rest, 10^5^/well) were cultured alone or together in 96-well plates with 1 nM IL-2 (n = 3). On day 5, cells were passaged to a 24-well plate with 1 nM IL-2. On day 9, cells were subjected to flow cytometric analysis of CD3 BV785 (SK7), CD4 BV421 (OKT4), CD8 Alexa Fluor 700 (SK1), CD25 FITC (M-A251), CD28 BV605 (CD28.2), CD74 PE (LN2), CD86 BV650 (FUN-1), CD134 PE-Cy5 (ACT35), and HLA-DR Spark Plus UV395 (L243). (A) Shown are representative dotplots of the Tcon-derived CD4^+^ JL3.T1 and JL3.C2 T-APC lines, the combination culture (JL3.C2 T-APCs + JL3 Tcons), and Tcons alone. (B) Mean MFI values are shown for CD4, DN, and CD8 gated cells. Statistical analyses were restricted to comparison of “Both” (i.e. JL3.C2/Tcon cocultures) versus Tcons alone to assess coculture-induced changes in the Tcon phenotype. (C) Cell yields (day 9) were assessed with CountBright Absolute Counting Beads to calculate cell/bead ratios. (D) Manual counting was also used to compare cell yields (day 9) relative to input values for T-APCs (10^4^/well) and Tcons (10^5^/well), given that DN and CD8^+^ single-positive Tcons comprised approximately 40% of cells by day 9. (E) Total cell yields based on manual cell counting were assessed on days 16, 20, and 24. (F) JL3.C2 T-APCs were cultured without TGF-β for 58 d and then split into parallel cultures with and without 1 nM TGF-β for 3 d. Cells were then tested in a 2-d proliferative assay ([^3^H]thymidine pulse during the last 24 h) in the presence of 1 nM IL-2 at 10^5^/well (200 µL in LymphoOne) in the presence or absence of 10 µg/mL functional grade anti-CD95 IgM mAb (EOS9.1). (G) CD4^+^ JL3.C2 T-APCs, CD8^+^ P3 T-APCs, SeAx T cells, and BW5147α/β^−^ T cells tested in a proliferative assay as described in panel F at 50,000 cells/well with designated dilutions of the EOS9.1 mAb. (E) Data were assessed with an unpaired 2-tailed *t* test. (C–F) Data were assessed with 1-way analysis of variance with a Tukey’s multiple comparisons test. (B, G) Data were assessed with a 2-way analysis of variance with Sidak’s or Tukey’s multiple comparison test. *P < 0.05, **P < 0.01, ***P < 0.001, ****P < 0.0001. CPM, counts per minute. Flow cytometric analyses showed that T-APCs activated resting conventional responder T cells, which subsequently acquired a T-APC phenotype. The activated responder T cells then eliminated the original T-APCs and later exhibited mutual killing, ultimately causing collapse of the culture. Additional experiments showed that T-APCs were highly susceptible to FAS-mediated cell death following treatment with an agonistic cross-linking anti-FAS IgM antibody.

This analysis also compared overall growth of activated T-APC/Tcon cocultures with T-APCs or Tcons cultured alone. By day 9, based on counting beads, yields of the combined T-APC/Tcon cultures were ∼32% of the yield for T-APCs alone (Fig. 10C, “T-APC alone” versus “Both”). When cultured alone, Tcons exhibited minimal yields and were largely dead. Based on manual counts (Fig. 10D), T-APCs expanded ∼26-fold in the presence of Tcons when compared with the 10^4^ starting population given that T-APCs comprised approximately 60% of these cultures. In contrast, T-APCs cultured alone expanded ∼188-fold. These data showed that the exponential growth of CD4^+^ T-APCs was inhibited by Tcon responders. In the presence of T-APCs, Tcons expanded ∼1.8-fold from the initiating population of 10^5^ cells. Notably, the presence of Tcons in the combined culture imposed long-term negative consequences on both populations (Fig. 10E). By day 16, the total number of cells in the combined culture was 16% that of T-APCs alone. By days 16, 20, and 24, the total yield of cells in the combined cultures was 8.8-fold, 54.2-fold, and 239.7-fold less than that of T-APCs alone due to a cessation of growth and extensive cell death of both populations in the T-APC/Tcon cocultures. The fratricidal collapse of the cocultures occurred despite the use of optimal cell culture conditions identical to the culture conditions that supported the exponential expansion of T-APCs that were cultured alone. These results suggest that T-APCs have regulatory activity by eliciting an initial phase of Tcon activation and a subsequent phase of activation-induced cell death.

Given the fratricidal collapse of FAS^+^ T-APC/Tcon cultures, a central question was whether FAS on T-APCs was functionally linked to cell death pathways. To address this issue, we used the anti-FAS IgM mAb EOS9.1 to crosslink FAS on JL3.C2 T-APC and elicit FAS signaling. The anti-FAS mAb elicited rapid loss of IL-2–dependent proliferation of JL3.C2 T-APCs coupled with visual observations of extensive cell death in 2-d cultures (Fig. 10F). Concentration dependence of anti-FAS–mediated killing revealed the sensitivity of the FAS-mediated cell death pathway (Fig. 10G). Notably, FAS-mediated killing was strongly influenced by TGF-β exposure, in that the TGF-β–cultured P3 T cells (CD8^+^) and JL3.C2 T-APCs (CD4^+^) T-APC were more resistant or more sensitive to the anti-FAS mAb, respectively. The anti-FAS mAb however had no adverse effect on SeAx and BW5147 control T cell lines. Overall, these data implicate the FAS pathway as a pivotal checkpoint for the T-APC subset and highlight the possibility that FAS may at least in part contribute to fratricidal outcomes in T-APC/Tcon cocultures.

## Discussion

This study reveals 4 unexpected observations including (a) the unique self-stimulatory qualities of human T-APCs, (b) the absence of any requirement for canonical professional APCs in sustaining T-APC growth, (c) the agonistic qualities of MHCII-restricted self-peptides presented by T-APCs, and (d) the agonistic activity of T-APCs in driving activation of polyclonal rested Tcons. First, although it is known that activated human T cells express MHCII glycoproteins to mediate T cell/T cell antigen presentation, this study provides evidence that a previously underappreciated professional T-APC subset utilizes a specialized auto-stimulatory mechanism of T-T agonistic autoantigen presentation to sustain long-term blastogenic T cell expansion in vitro. Second, although professional APCs can be used to drive AMLR-like proliferation, our results demonstrate that AMLR-driven continuous T-APC lines can be sustained in the absence of canonical professional APCs such as DCs, macrophages, and B cells. Thus, T-APCs have the full spectrum of signaling qualities needed to drive sustained, AMLR-like proliferation in long-term T cell cultures. Third, while it is established that antigens can act as full agonists, partial/mixed agonists/antagonists, or full antagonists for particular clonotypic T cell antigen receptors,^73–76^ this study is unique in providing evidence that endogenous self-peptides can act as strong agonists capable of driving exponential blastogenic AMLR-like T cell expansion. Fourth, although it is known that T cells can present antigen to rested conventional T cell responders, this study uniquely provides evidence that a professional T-APC subset can efficiently activate a polyclonal Tcon responder population to adopt an activated T-APC phenotype. Overall, this study reveals a T-APC entity that is unanticipated by contemporary models of immune regulation. A reasonable inference is that T-APCs are specialized to hone self-reactivity of the TCRα/β repertoire, which is needed for optimal antigen recognition and regulatory/conventional T cell balance.

### T-APCs utilize a unique autostimulatory mechanism

The expression of MHCII glycoproteins on activated T cells is well appreciated in the field. The prevailing view is that antigen presentation by activated T cells plays a secondary role compared with professional APCs (e.g. B cells, DCs, and macrophages) by supporting recognition of foreign antigen during host defense. T-APCs are considered redundant or subordinate to professional APCs because T-APC activity is thought to be transient and limited to a temporary phase of T cell activation. This study however challenges this paradigm and instead implicates T-APCs as a unique professional APC subset that merges the cardinal activities of professional APCs and T cells. The observation that T-APCs constitutively exhibit high levels of MHCII and costimulatory molecules and present “agonistic self” over 1 to 2 mo of continuous culture supports the view that T-APCs qualify as a specialized subset of professional APCs that synthesize and respond to stimulatory self-antigens. T-APCs are constituents of the CD4^+^ and CD8^+^ T cell lineages and comprise distinct subsets that feature bimodal variations in CD25, CD69, and PD1 expression. T-APCs arise from low-frequency precursors and eventually emerge as exponentially expanding populations after a 1- to 2-wk period of static culture. Thus, clonal expansion of rare progenitors, unfolding differentiation programs, and/or competitive niche development may be required before the subsequent onset of T-APC outgrowth. T-APCs may require selective environmental conditions, including those that drive lymphopenic homeostatic expansion or AMLRs. Because continuous T-APC lines were reliably derived from over 18 normal healthy human donors, one can conclude that T-APCs are intrinsic to a normal immune system and are not an idiosyncrasy of a particular individual.

The main drivers of continuous CD4^+^ T-APC growth appeared to be agonistic self-pMHC complexes that were recognized by self-reactive TCRα/β in an auto-stimulatory loop (Figs. 3–5). The evidence is summarized by the following observations. The neutralizing RPA-T4 anti-CD4 mAb inhibited IL-2–driven growth, whereas the non-neutralizing OKT4 anti-CD4 mAb lacked inhibitory activity. The RPA-T4 and OKT4 mAbs exhibited allosteric interactions, such that the inhibitory activity of the RPA-T4 mAb was reversed by the OKT4 mAb. The RPA-T4/OKT4 interplay provides evidence that the unimpeded D1 CD4 domain was critical for agonistic activity. Notably, the CD4 D1 domain is known to interact with the β2 domain of MHCII glycoproteins to mediate TCR-MHCII interactions.^77–81^ The RPA-T4 mAb inhibited CD4^+^ but not CD8^+^ T-APC growth, and this inhibitory action was evident only in culture conditions featuring extensive cell-cell contact and cell clustering. The RPA-T4 inhibitory mechanism affected IL-2 responsiveness rather than IL-2 bioavailability and shifted the CD25^high^/CD25^low^ balance in favor of the CD25^low^ subset. Because a neutralizing anti-CD4 mAb inhibits IL-2 stimulated CD4^+^ T-APC growth, self-pMHCII-TCRα/β-CD4 complexes are implicated as intermediates in sustaining IL-2 reactivity.

Self-stimulatory T-APC activity was also tested by assaying expression of CD40L because TCR signaling, rather than costimulatory or cytokine signaling, is known to be the main instigator driving de novo CD40L expression during T cell activation.^63^^,^^64^^,^^68^ T-APC–mediated self-stimulatory activity was associated with constitutive expression of CD40L by a mechanism that was inhibited with an anti-HLA-DR mAb. These data indicate that T-APC self-stimulatory activity is a TCR-driven engagement because no other costimulatory and cytokine pathway has been described that can initiate and sustain CD40L expression on T cells in perpetuity. Thus, CD4 and MHCII are implicated as key molecules underpinning continuous CD4^+^ T-APC expansion.

### TGF-β was needed for long-term viability of T-APC lines

T-APC lines that were derived from freshly isolated PBMCs required two exogenously added cytokines for stable long-term expansion. The prototypic Treg-associated cytokine IL-2 was the primary growth factor. The Treg-associated differentiation factor TGF-β was also required to sustain long-term expansion (Figs. 1, 2, 7). TGF-β in synergy with TCR engagement is well known to elicit differentiation of FOXP3^+^ Tregs.^43^^,^^49^ Because FOXP3 mitigates reactivation induced cell death,^82–85^ a TGF-β/FOXP3 axis (Fig. 7) may help alleviate activation-induced cell death to enable efficient T-APC expansion. TGF-β also augmented MHCII and CD25 expression in CD25^high^ T-APCs (Fig. 1). These data are consistent with the interpretation that TGF-β augmented higher levels of self-pMHCII expression, which in turn caused higher levels of agonistic TCR engagement, followed by CD3 downregulation, heightened CD25^high^ expression, greater IL-2 responsiveness, increased FOXP3 expression, and FOXP3-mediated resistance to reactivation induced cell death (Fig. 1) .^83^ A previous study on mitogen-stimulated human PBMC cultures showed that TGF-β elicited higher HLA-DR expression as measured by increased percentages of HLA-DR^+^ T cells and higher HLA-DR MFI values on a per-cell basis in association with higher FOXP3^+^ T cell accumulation.^86^ TGF-β also increased PD1 expression in various T-APC lines, particularly in CD25^low^ T-APCs during long-term expansion. PDL2-PD1 interactions elicit PD1-mediated SHP-2 tyrosine phosphatase recruitment that inhibits proximal TCR signaling and blocks CD28 costimulatory signaling.^87^^,^^88^ However, PD1 does not invariably cause T cell nonresponsiveness. Depending on the strength and chronicity of antigenic/inflammatory signals, transient PD1 expression can mitigate excessive T cell signaling to protect T cells from overactivation and prevent activation-induced cell death, thereby preserving T cells in a viable but subresponsive state. Overall, TGF-β may act through multiple stimulatory (HLA-DR, CD25) and modulatory (PD1) pathways to impose homeostatic boundaries to optimize T-APC expansion at different TCR/IL-2 signaling intensity bandwidths; unbridling full TCR responsiveness in low-intensity environments while balancing lower TCR responsiveness over cell death in high-intensity signaling environments. This TGF-β “buffer” may in part explain why TGF-β–supplemented cultures exhibit long-term autologous growth, whereas T-APCs cultured in the absence of TGF-β eventually lose viability (Figs. 2, 7). However, mechanisms underpinning TGF-β–dependent pathways are not understood, and other undefined TGF-β–dependent mechanisms may also regulate T-APC expansion.

### Distinct CD25^high^ and CD25^low^ T-APC subsets were revealed by low-zone versus high-zone cytokine signaling

Cytokine concentrations used in this study (e.g. routine passages starting 1 nM IL-2 and 1–10 nM TGF-β) were chosen to optimize physiological responses, given that both cytokines are rapidly consumed during multiday passages that often yielded ∼10^6^ cells/mL. Both IL-2 and TGF-β receptor systems are consumptive receptor systems that internalize receptor/ligand complexes and degrade ligands in acidified endosomal compartments. TGF-β ligand–receptor complexes can be routed via caveolae-mediated endocytic pathways for intracellular degradation, whereas TGF-β–internalized TβRI/TβRII receptors may also exhibit multiple rounds of signaling via Rab11-dependent recycling to the cell surface.^89^^,^^90^ Likewise, the IL-2–IL-2Rαβγ complex is rapidly endocytosed wherein IL-2 is trafficked for degradation in an acidified endosomal-lysosomal compartment and CD25 is repetitively recycled to the cell surface.^91–93^ Thus, cytokine concentrations were chosen to ensure survival and growth of T cells inclusive of both CD25^high^ and CD25^low^ T-APC subsets throughout each passage. Given that the dissociation constant of high-affinity IL-2Rαβγ (1–10 pM) is considerably more sensitive than that for the low-affinity IL-2Rβγ (0.5–5 nM),^94^ a 1 nM IL-2 concentration was adequate for stimulation of both CD25^high^ and CD25^low^ T-APC subsets at culture initiation, whereas subsequent IL-2 consumption likely favored dominance of the CD25^high^ subset toward the end of each passage. Conversely, high IL-2 concentrations (≥10 nM) favored dominance of CD25^low^ T-APCs. Thus, IL-2 concentrations that favored outgrowth of CD25^high^ versus CD25^low^ subsets provided a benchmark for T cell perception of IL-2 signaling efficiencies and provided insight into relationships linking initial cytokine concentrations, receptor affinities, and ultimate outcomes (Figs. 8, 9).

At low IL-2/TGF-β concentrations, T-APCs exhibited a canonical CD25^high^ CD69^low^ PD1^low^ CTLA4^+^ phenotype marked by a robust IL-2 signature including larger cell size, elevated expression of canonical Treg-associated markers (FOXP3, CTLA4, LAG3, GITR), T-APC markers (HLA-DR, CD86, CD80), and CD28 and CD127 downregulation (Figs. 8, 9). In contrast, high concentrations of IL-2 and TGF-β elicited bifurcation of two major T-APC subsets via dominant outgrowth of a CD25^low^ CD69^+^ PD1^+^ CTLA4^low^ subset. Thus, the CD69 and PD1 markers qualitatively revealed distinct bimodal T-APC populations. Both proteins mediate important roles in immune regulation. CD69 antagonizes S1PR1 to inhibit leukocyte egress from secondary lymphoid organs.^95–99^ Similarly, CD69 favors residency of tissue-resident memory T cells. Distinct CD69^high^/CD69^low^ subsets have been noted in other studies with the implication that CD69^+^ FOXP3^+^ Tregs have robust suppressive function^100–102^ and play a pivotal role in regulatory T cell biology.^103^^,^^104^ PD1 is a checkpoint inhibitor that increases TCR/CD28 stimulation thresholds, thereby requiring higher stimulus intensities for productive T cell responses.^105–107^ Consistent with data shown in Figures 8 and 9, previous studies have indicated that TGF-β activates SMAD3 which binds to the *PDCD1* promoter to augment PD1 transcription.^108^^,^^109^ Given the high concurrence of CD69 and PD1 coexpression in the CD25^low^ subset, one would surmise that CD69 and PD1 mediate coordinate functions during tissue residency in which intrinsic high-efficiency APC activity and high tissue-specific antigen concentrations yield high TCR stimulus intensities.

An attractive model is that CD4^+^ and CD8^+^ lineages include T-APC subsets that have TCR responsiveness optimized for different bandwidths of stimulus intensity. The CD25^high^ subset expresses CD25 and lacks PD1, thereby conferring high sensitivity to low concentrations of IL-2, TCR, and costimulatory signals. Conversely, the CD25^low^ subset lacks CD25 and expresses PD1, thereby conferring lower sensitivity to high concentrations of IL-2, TCR, and costimulatory signals. Thus, CD25^high^ and CD25^low^ T-APC bandwidths may provide complementary coverage of productive T-APC function in low or high stimulus environments, respectively. Differential CD69 expression provides suggestive evidence that CD25^high^ and CD25^low^ T-APCs occupy unique physical niches, where CD69^low^ T-APCs may mediate recirculatory surveillance and CD69^high^ T-APCs may mediate residential surveillance in peripheral tissues. A likely possibility is that TGF-β facilitates a developmental pathway, eliciting initial differentiation of CD25^+/−^ T-APC precursors to a CD25^high^ PD1^−^ phenotype (Fig. 1), which is poised in the presence of high TGF-β/IL-2 concentrations to further differentiate into a CD25^low^ PD1^+^ phenotype (Figs. 8, 9). Thus, TGF-β signaling intensity may help prime T-APCs for adaption to local environments.

### T-APCs share significant attributes with professional APCs, FOXP3^+^ Tregs, and Tfr T cells

This study reveals an unexpected piece of the immunological puzzle, describing a unique TCRα/β T cell entity that has attributes of professional APCs and canonical Treg/Tfr subsets (Table 3). For example, CD25^high^ T-APCs and canonical Tregs share several commonalities, including agonistic reactivity to self-pMHCII complexes; expression of a FOXP3^+^, CD25^high^, and CD127^low^ phenotype; and dependence on IL-2 and TGF-β for growth and differentiation. T-APCs also share several commonalities with the Tfr Treg subset, including expression of the transcription factors FOXP3 and BCL6 together with high expression of CD278/ICOS. A defining feature of Tfr cells is the coexpression of FOXP3 with Tfh-associated markers such as BCL6, ICOS, and PD-1.^110–112^ T-APCs exhibited regulatory activity by activating Tcons, which acquired cytotoxic effector activity that eliminated the originating T-APCs, followed by the widespread fratricide of activated APC-like Tcons over 3 wk (Fig. 10). Likewise, previous studies have reported the coupling of cytotoxic T-APC activity with fratricidal T cell to T cell killing.^113–115^ These findings suggest that some regulatory mechanisms associated with T-APC function be unique from those classically associated with canonical Tregs or Tfr cells. The mechanism of T-T fratricide is currently unknown, although the FAS/FAS-L system appears poised to play a major role (Fig. 10). T-APCs also express high levels of CD123/IL3Ra, which is used to distinguish human plasmacytoid DCs from classical myeloid DCs.^72^ It is unknown whether these commonalities reflect lineage/subset overlap or convergent environmental adaptation.

**Table 3:** Phenotypic relationships of T-APCs with professional APCs, Tregs, and Tfrs.

Cell type	Shared markers between cell type and T-APC subset	Relevant T-APC subset
**Myeloid DCs**	MHCII^high^, CD74, CD80, CD86	CD25^high^ & CD25^low^
**Plasmacytoid DCs**	MHCII^+^, CD74, CD123/IL3Rα	CD25^high^ & CD25^low^
**B cells**	MHCII^high^	CD25^high^ & CD25^low^
**T cell lineage**	TCRα/β, CD3, CD4, and CD8 lineages	CD25^high^ & CD25^low^
**T cell activation**	CD134, CD40L	CD25^high^ & CD25^low^
**Tregs**	CD25^high^, CD127^low^, FOXP3, CTLA4	CD25^high^
**Tfrs**	FOXP3, BCL6, ICOS	CD25^high^ & CD25^low^
**Tfrs**	CD69, PD1	CD25^low^
**Regulatory**	PD1, PDL2, GITR, LAG3, CD95/FAS, CD178^low^	CD25^high^ & CD25^low^

### Clinical applicability

It is intriguing to consider the potential of T-APCs for cell-based therapy given that human T-APCs constitutively express high levels of FOXP3 and mediate regulatory function. Several advantages are notable compared with contemporary approaches for Treg-based cell therapy. T-APCs do not require cell purification because T-APCs can be expanded directly from nonfractionated PBMCs based on superior cell expansion kinetics. The cell expansion strategy is dependent upon simple ingredients including xeno-free media, IL-2, and TGF-β. T-APCs do not require genetic engineering to confer self-reactivity, stable FOXP3 expression, or exponential growth. T-APC cultures yield exponential growth for 1 to 2 mo without T cell exhaustion. Required equipment includes standard tissue culture flasks and CO_2_ incubators. Theoretically, T-APCs expanded in vitro and transferred back into a syngeneic host would predictably have robust in vivo survival because the relevant TCRs are preselected to have homeostatic self-reactivity. The main limitation at this time is that we do not understand the regulatory role of T-APCs in immune homeostasis in vivo.

## Data Availability

All data supporting the major conclusions of this study are included in this article: raw data files are available from the corresponding author upon reasonable request.
